# Surround Inhibition Mediates Pain Relief by Low Amplitude Spinal Cord Stimulation: Modeling and Measurement

**DOI:** 10.1523/ENEURO.0058-22.2022

**Published:** 2022-10-04

**Authors:** John E. Gilbert, Nathan Titus, Tianhe Zhang, Rosana Esteller, Warren M. Grill

**Affiliations:** 1 Department of Biomedical Engineering, Duke University, Durham, NC, 27708; 2 Neuromodulation Research and Advanced Concepts, Boston Scientific Neuromodulation, Valencia, CA, 91355; 3 Department of Electrical and Computer Engineering, Duke University, Durham, NC, 27708; 4 Department of Neurobiology, Duke University School of Medicine, Durham, NC, 27708; 5 Department of Neurosurgery, Duke University School of Medicine, Durham, NC, 27708

**Keywords:** computational modeling, *in vivo* recording, neuropathic pain, spinal cord stimulation

## Abstract

Low-frequency (<200 Hz), subperception spinal cord stimulation (SCS) is a novel modality demonstrating therapeutic efficacy for treating chronic neuropathic pain. When stimulation parameters were carefully titrated, patients experienced rapid onset (seconds–minutes) pain relief without paresthesia, but the mechanisms of action are unknown. Using an integrated computational model and in vivo measurements in urethane-anesthetized rats, we quantified how stimulation parameters (placement, pulse width, frequency, and amplitude) influenced dorsal column (DC) axon activation and neural responses in the dorsal horn (DH). Both modeled and recorded DC axons responded with irregular spiking patterns in response to low-amplitude SCS. Maximum inhibition of DH neurons occurred at ∼80% of the predicted sensory threshold in both modeled and recorded neurons, and responses were strongly dependent on spatially targeting of stimulation, i.e., the complement of DC axons activated, and on stimulation parameters. Intrathecal administration of bicuculline shifted neural responses to low-amplitude stimulation in both the model and experiment, suggesting that analgesia is dependent on segmental GABAergic mechanisms. Our results support the hypothesis that low-frequency subperception SCS generates rapid analgesia by activating a small number of DC axons which inhibit DH neuron activity via surround inhibition.

## Significance Statement

Spinal cord stimulation (SCS) is an effective treatment from chronic pain, but conventional stimulation generates paresthesias, a buzzing sensation that some patients find uncomfortable. Recent studies have demonstrated substantial pain relief using low-frequency SCS that does not generate paresthesia; however, it is unclear how this form of stimulation works. In this study, we used computational models and recordings of dorsal horn (DH) neurons and dorsal column (DC) axons to study low-frequency, low-amplitude SCS and proposed a novel mechanism of action. The mechanism of action we proposed may help design future parameter selection and drive the development of SCS as a therapy.

## Introduction

Spinal cord stimulation (SCS) is an established treatment for chronic pain. SCS was developed based on the gate-control theory, which posits that activation of large diameter (Aβ) afferents leads to inhibition of pain transmitting neurons in the dorsal horn (DH; Melzack and Wall, 1965). Conventional SCS uses low-stimulation frequencies (<200 Hz) at amplitudes above perception threshold (PT) to activate dorsal column (DC) axons and inhibit pain (Parker et al., 2012). Stimulation above PT generates paresthesias, artificial sensations that some patients find undesirable. Some subperception modalities of SCS – using higher stimulation frequencies (1–10 kHz) – demonstrated at least equivalent pain relief without evoking parethesias, but the timescale for pain-relief is generally slower than with conventional SCS, suggesting a different mechanism of action (Al-Kaisy et al., 2014). A notable exception was low-frequency stimulation delivered below PT that resulted in rapid onset of pain relief, and efficacy appeared to require specific stimulation parameters and precise spatial targeting of stimulation (Metzger et al., 2021).

Although significant and sustained pain relief was reported by patients, the mechanism(s) of action for low-frequency subperception SCS remained unclear. We surmised that the mechanism was based on activation of DC axons since it is applied at conventional frequencies and requires that electrodes are configured to achieve pain-paresthesia overlap with above-perception stimulation amplitudes during device programming. Further, the precise spatial targeting suggested that the mechanism may depend on activation of specific axons based on the somatotopic organization within the dorsal columns (Smith and Bennett, 1987; Feirabend et al., 2002). Therefore, we tested the hypothesis that surround inhibition contributed to the mechanisms of low-frequency subperception SCS. Surround inhibition refers to sensory networks where spatial selectivity is amplified by the contrast of excitation from inputs within the central receptive field and inhibition from inputs in the surround receptive fields (Blakemore et al., 1970; Beck and Hallett, 2011). The importance of surround inhibition to sensation and pain is exemplified by the expansion of receptive field areas following disinhibition and inhibition of DH neurons from electrical stimulation, including by SCS, of sensory fibers from adjacent receptive fields (Hillman and Wall, 1969; Menétrey et al., 1977; Kato et al., 2009, 2011; Luz et al., 2010; Lee et al., 2019; Fan and Sdrulla, 2020).

We combined validated computational models and *in vivo* recordings from DC axons and DH neurons to study the mechanisms underlying low-frequency subperception SCS. DC axons responded to stimulation at amplitudes below putative sensory threshold, but patterns of activation were asynchronous at amplitudes close to the activation threshold (AT) both *in silico* and *in vivo*. Models predicted that such DC axon activity produced inhibitory effects in the DH network, and that maximum suppression of DH neurons occurred when low amplitude electrical stimulation was delivered to the surround receptive field. Experimental measurements of the effect of Aβ electrical stimulation (Aβ-ES) on activity of DH neurons corroborated model predictions, and partial abolition of inhibitory effects by the local application of bicuculline further supported the presence of a segmental inhibitory mechanism consistent with depictions of surround inhibition (Hillman and Wall, 1969). These results support that the pain-relieving effects of low-frequency, fast-acting subperception SCS are mediated by segmental surround inhibition and raise the possibility that surround inhibition can be exploited to optimize SCS.

## Materials and Methods

### Code accessibility

Code required to reproduce the figures in this manuscript will be made available. Model code is posted on ModelDB.

### Model of dorsal column axon activation

**Figure 1.**
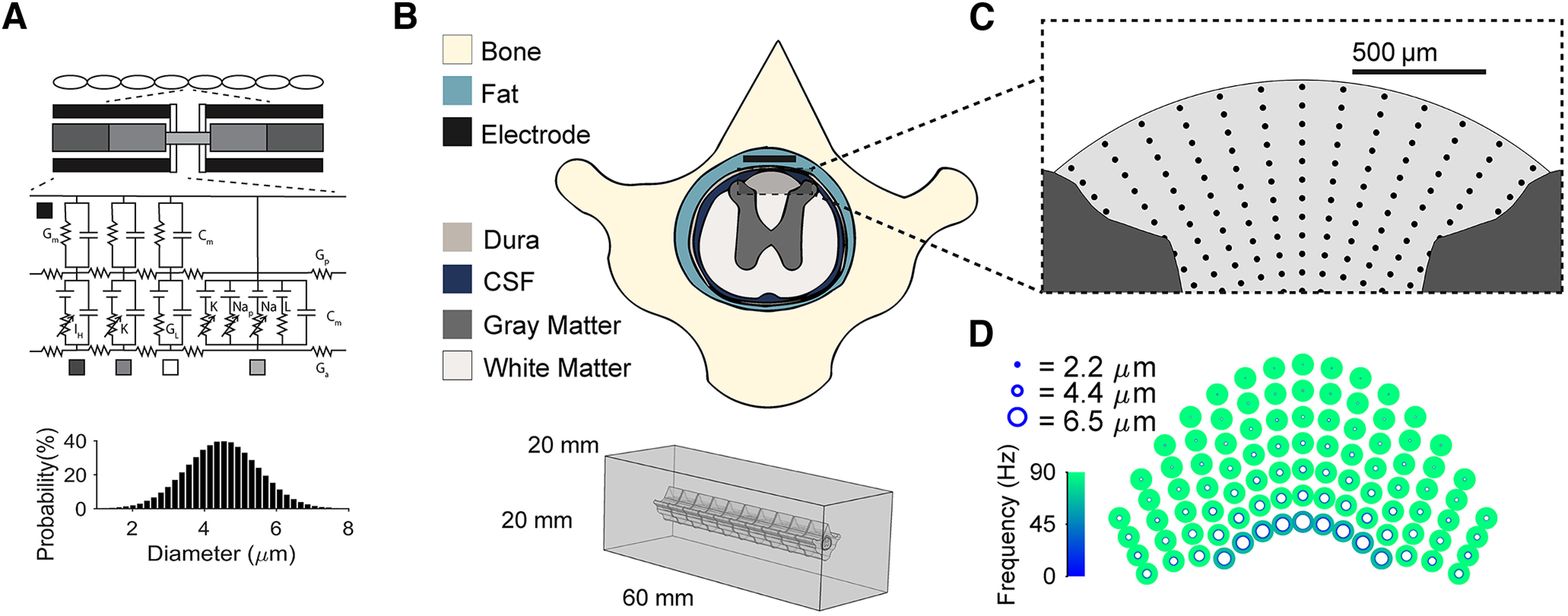
Modeling dorsal column (DC) axon responses to spinal cord stimulation. ***A***, Individual DC axons were modeled using a modified MRG model axon (McIntyre et al., 2002). Axon diameters were selected from a normal distribution with mean of 4.4 μm and a SD of 1 μm. ***B***, A previously published finite element method model of the rat spinal cord was used to calculate the electric potentials at the model axon compartments during SCS (Pelot et al., 2018). ***C***, Axons with diameters from 2 to 8 μm were modeled in the DCs at the locations shown with black dots. ***D***, Example responses of DC axons in all positions and all diameters at 60% MT/60 μA. Only activated axons are shown and the color of each axon represents its firing rate in response to symmetric biphasic rectangular 90-Hz, 275-μs stimulation.

We modeled DC axons using cable models of mammalian axons modified to account for dorsal column axon membrane dynamics and responses to stimulation (Fig. 1*A*; McIntyre et al., 2002; Titus et al., 2022). We coupled the DC axon models to a previously described finite element model (FEM) of the rat spinal cord (Pelot et al., 2018) to quantify responses to a range of stimulation amplitudes, pulse durations and pulse repetition frequencies (Fig. 1*B*). In brief, the FEM was built using transverse MRIs at the thoracic level (T10) and consisted of 10 vertebrae positioned within a box of 20 × 20 × 60 mm with outer boundaries grounded. Mesh resolution was doubled until activation thresholds changed by <1% (∼3.14 × 10^6^ elements). We positioned in the epidural space a model of our *in vivo* electrode: two 1 × 2 mm platinum contacts spaced 2 mm apart center-to-center along the length of the spine. The conductivities of the tissues and materials were selected from literature (Pelot et al., 2018). The potentials generated by SCS were extracted from the FEM and applied to the compartments of the validated DC axon model. Axon positions were selected to sample the range of positions within the DC, and axon diameters ranged from 2.2 to 6 μm with an increment of 0.2 μm and from 6 to 8 μm with an increment of 0.5 μm. The stimulation geometry was a simple bipolar configuration, with one contact set as the cathode and the other contact set to an equal anode; a single point current source was placed inside each electrode contact, and the outer boundary surfaces of the model were set to ground. All stimulation pulses were symmetric, biphasic, and rectangular. Simulated amplitudes ranged from 20 to 150 μA, except for 10-kHz stimulation (Extended Data Fig. 2-1), and simulated amplitudes for 10-kHz stimulation were 150 and 300 μA based on a predicted MT of 300 μA.

### Network models of the dorsal horn

**Figure 2.**
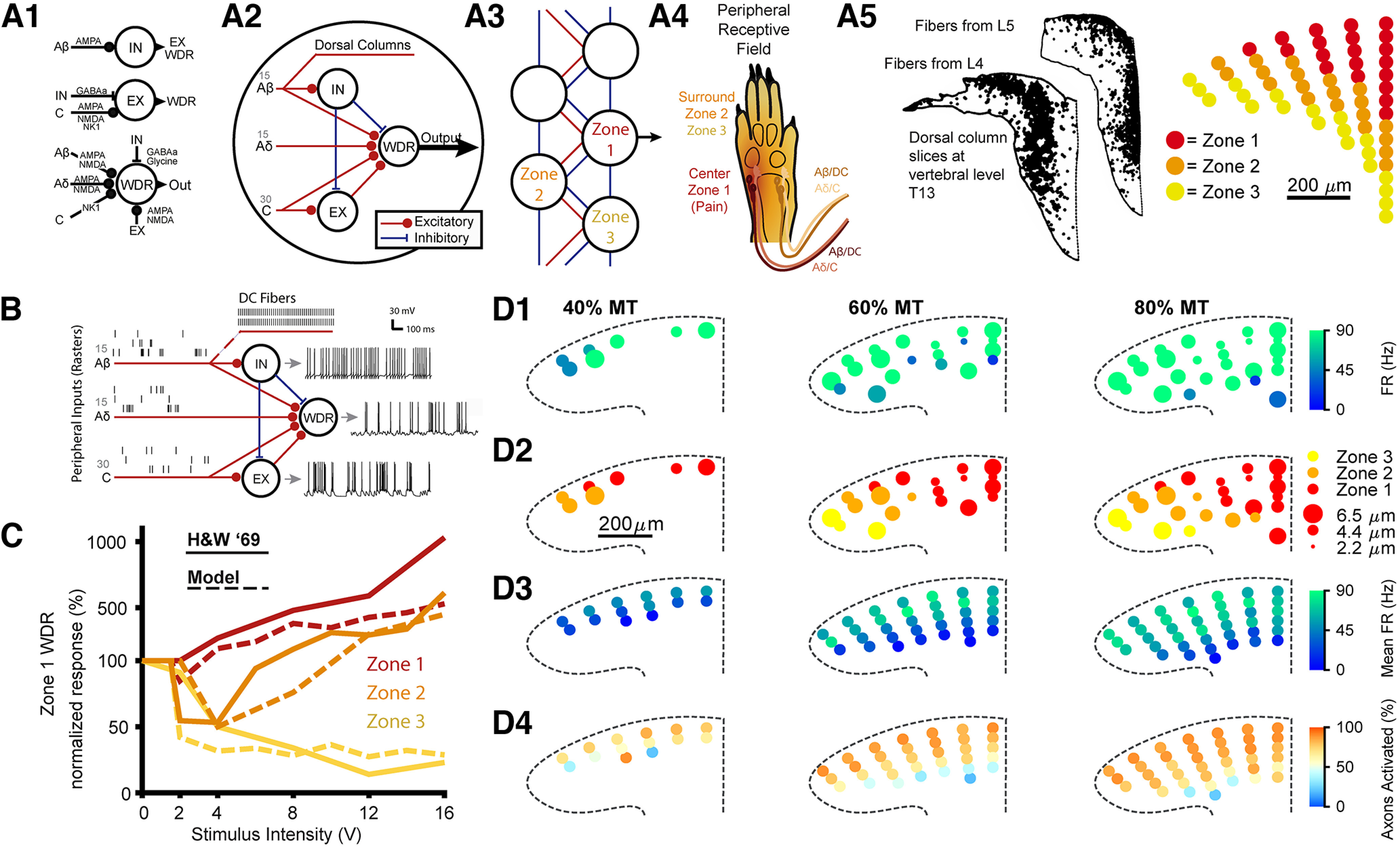
Modeling DC axon and dorsal horn (DH) network responses. ***A1***, Synaptic connections between neurons for a single node in the model. ***A2***, Neural connections between peripheral afferent fibers, DC axons, inhibitory and excitatory interneurons, and a WDR projection neuron within a single node of the DH model. ***A3***, Connections between nodes in the multinodal circuit model of the DH. Inhibitory and excitatory connections between nodes are from local interneurons to WDR neurons in the other nodes. ***A4***, Representation of center and surround in peripheral receptive field. Center axons represent the pain area (zone 1), while surround is split into near surround (zone 2), and far surround (zone 3). ***A5***, Representation of center and surround in the DCs. Axons from the L5 nerve root are most medial and dorsal at the T13 vertebral segment (Smith and Bennett, 1987) and axons from the surrounding area from the L4 nerve root are positioned ventrally and laterally from the center fibers. Axons in the model were assigned to different zones based on their position within the DCs. ***B***, Example peripheral afferent inputs and neuron responses. Peripheral afferent spike trains representing pain inputs applied to the model through the Aβ inputs are shown on the left. Transmembrane voltage traces for each model neuron are shown on the right. See Extended Data Figure 2-1 for example responses of WDR neurons over time during the simulations. ***C***, Response of center model WDR neuron to stimulation in each receptive field compared with experimental recordings of Lamina V WDR neurons. Increasing amplitude was modeled as increasing afferent axon recruitment in each peripheral zone based on normalized recruitment curves (Sdrulla et al., 2015). Solid lines represent the experimental responses while dashed lines represent model responses. The *y-*axis is linear below 100% and log above 100%. ***D1***, Individual DC topography showing an example of DC activation, i.e., inputs to the network model at different amplitudes. Circle size represents axon diameter and circle color represents axon firing rate. ***D2***, The position and corresponding zone of each activated axon with parameters from ***D1***. ***D3***, Average firing rate for all positions across 25 randomized samples DC topography. ***D4***, Percent of axons activated in each position across 25 samples of DC topography. See Extended Data Figure 2-1 for DC axon responses to kilohertz frequency stimulation.

We developed a biophysical network model of DH neurons based on a prior model and features of the model were described previously (Fig. 2*A1*; Zhang et al., 2014). The model was adapted to account for distributed center-surround network dynamics. The model had three types of neurons, an inhibitory (IN) interneuron, an excitatory (EX) interneuron, and a wide-dynamic range (WDR) projection neuron, and each model neuron contains four compartments: a dendrite, soma, axon hillock, and axon (see Table 1 for neuron geometries). We modeled ionic currents in each type of model neuron using Hodgkin–Huxley-like membrane models replicated from the prior model (Zhang et al., 2014). Each model node contained one of each neuron, 15 Aβ fiber inputs, 15 Aδ fiber inputs, and 3°C-fiber inputs. We modeled neuropathic pain inputs to the model as spike times along these afferents with mean firing rates (A-fiber μ = 2.2 Hz, C-fiber μ = 1.5 Hz) drawn from recordings of afferents from a neuroma (Wall and Gutnick, 1974). We also added bursting (μ_isi_intraburst_ = 30 ms, μ_isi_interburst_ = 551 ms, μ_spikes_per_burst_ = 6) in one third of the A-fibers (Kajander and Bennett, 1992; Liu et al., 2000). In the model, each zone was made up of an individual node (Fig. 2*A2*) with distinct inhibitory and excitatory connections between nodes (Fig. 2*A3*), and each zone in the model represented a corresponding peripheral receptive field area (Fig. 2*A4*) and DC somatotopy (Fig. 2*A5*). One zone was designated the “center” zone (“zone 1”), and flanking zones (“zone 2” and “zone 3”) representing the “surround” were added and reciprocally interconnected via excitatory and inhibitory connections (Fig. 2*A3*). Connections between zones were from the excitatory or inhibitory interneurons in one node (e.g., zone 2) to the WDR neuron in another zone (e.g., zone 1). Synaptic connections between individual neurons replicated the connections from the previous model and Table 2 displays the synaptic connections between neurons. Synaptic properties also matched the prior model (Table 3). We validated the DH network model by comparing zone 1 model WDR neuron responses to experimental recordings of Lamina V WDR neurons in response to increasing amplitude of peripheral electrical stimulation (Hillman and Wall, 1969). The activity of the zone 1 model WDR neuron matched the magnitude and pattern of responses of experimentally recorded neurons during simulated peripheral nerve stimulation (Fig. 2*C*), indicating that the model architecture replicated realistic center-surround receptive field dynamics. All simulations were conducted in the NEURON simulation environment (v7.5 and v7.6) using second-order implicit Crank–Nicholson integration and a timestep of 0.0125 ms (Hines and Carnevale, 1997). Each simulation was run for 18 s with stimulation on for 10 s at the end.

**Table 1:** Neuron geometries for individual compartments

Neuron	Dendrite (cylinder [diameter, length])	Soma (sphere [diameter])	Axon hillock (cone [initial diameter, final diameter, length])	Axon (cylinder [diameter, length])
EX	[3 μm, 300 μm]	[25 μm]	[2 μm, 1 μm, 9 μm]	[1 μm, 1000 μm]
IN	[3 μm, 400 μm]	[10 μm]	[1 μm, 0.5 μm, 30 μm]	[1 μm, 1000 μm]
WDR	[2.5 μm, 350 μm]	[20 μm]	[2 μm, 1 μm, 9 μm]	[1 μm, 1000 μm]

**Table 2:** Synaptic connections and conductances between neurons

Source	Target	Synapse type	Maximum conductance (g_max,_ nS)
Aβ	IN	AMPA	14.6
Aβ	WDR	AMPA	24
Aβ	WDR	NMDA	0.1
Aδ	WDR	AMPA	24
Aδ	WDR	NMDA	0.1
C	EX	AMPA	8
C	EX	NMDA	4
C	EX	NK1	0.02
C	WDR	NK1	0.014
IN	EX	GABA_A_	5.3
IN2*	EX1*	GABA_A_	3.66
IN3*	EX1*	GABA_A_	3.66
IN	WDR	GABA_A_	5.3
IN	WDR	Glycine	5.3
IN2*	WDR1*	GABA_A_	4.5
IN2*	WDR1*	Glycine	4.5
IN3*	WDR1*	GABA_A_	4.5
IN3*	WDR1*	Glycine	4.5
EX	WDR	NMDA	0.21
EX	WDR	AMPA	0.29
EX2*	WDR1*	NMDA	0.014
EX2*	WDR1*	AMPA	0.14

Starred neurons(*) show connections between nodes and these connections are reciprocal and repeated for each node (see Fig. 2).

**Table 3:** Synaptic time constants and reversal potentials

Synapse	Rise time constant, τ_1_ (ms)	Fall time constant, τ_2_ (ms)	Reversal potential, E_syn_ (mV)
AMPA	0.1	5	0
NMDA	20	100	0
NK1	100	3000	0
GABA_a_	0.1	20	−70
Glycine	0.1	10	−70

10.1523/ENEURO.0058-22.2022.f2-1 Extended Data Figure 2-1 Low-rate but not kilohertz frequency stimulation produces rapid-onset inhibition of DH neurons. ***A***, Filtered firing rate of model WDR neurons. Each light line represents an individual response with a different map of randomly selected dorsal column axons. Dark lines represent median response. ***B***, DC axon responses to kilohertz frequency stimulation. Light lines represent individual axon responses and dark line represents the median response. MT was estimated as 100 μA for 1200-Hz/135-μs stimulation and 300-μA/30-μs stimulation based on *in vivo* measurements in rats. ***C***, Filtered firing rate of model WDR neuron responses to kHz frequency stimulation. ***D***, Timescale of example trial from *in vivo* recordings of spontaneous activity (0–30 s) followed by Aβ-ES (30–60 s). Each line represents the filtered firing rate of an individual neuron. Colors represent the mean normalized change in firing rate across the 30-s window. Download Figure 2-1, TIF file.

We compiled a library of DC axon responses for each stimulation configuration with the responses of all possible axon position and diameter combinations. As dorsal column fibers are generally collaterals of large myelinated primary afferents (Melzack and Wall, 1965; Niu et al., 2013), individual DC axon responses were sampled from the library of responses to generate spike time inputs to the DH network model via the model’s Aβ inputs. Each individual fiber was assigned as an input to a node of the network model based on its medial-lateral and dorsal-ventral position within the DCs. According to anatomic tracings of DC axons, fibers reach their most dorsal and medial point approximately two levels rostral to their entry point into the spinal cord (Smith and Bennett, 1987; Niu et al., 2013). From there, fibers are pushed laterally as new fibers enter the cord and preserve their relative somatotopic organization up to the DC nuclei (Loutit et al., 2021; Smith and Bennett, 1987). For center fibers, we selected the 20 most medial and dorsal fiber positions by minimizing the equation *Z* = *X*^0.15^ + *Y*^0.45^ where *X* was the medial-lateral position and *Y* was the rostral-caudal position. We randomly selected 15 of these positions for each simulation as the primary target of stimulation. When stimulation targeted the center of the receptive field in the model, all 15 axons were inputs to zone 1. When stimulation targeted the surround, all 15 axons were inputs to zone 2. When stimulation targeted a mix of center and surround, eight fibers were inputs to zone 1 and seven fibers were inputs to zone 2. We repeated this process and 15 of the 20 next most medial and dorsal fiber positions were assigned to the secondary target of stimulation. When stimulation targeted the center receptive field area, these fibers corresponded to zone 2 inputs. Finally, we assigned 15 of the next 20 fibers as inputs to zone 3, representing the distant surround. For each fiber position, we randomly selected a fiber diameter from a range of diameters commonly found within the rat DCs (μ = 4.4 μm, σ = 1.0 μm).

Using the procedure described above, we generated 25 maps of activated DC axons to test stimulation inputs while accounting for biological variability with different fiber diameters and positions. We quantified the response of each specific axon across amplitudes for each combination of frequency and pulse width (Fig. 2*D1*) and assigned each DC fiber as an input to the DH network based on the fiber position and spatial targeting condition (Fig. 2*D2*). Across all 25 maps of DC axons, the mean firing rate of the sampled axons decreased for axons further away from the electrode (Fig. 2*D3*), and axons in the most dorsal positions close to the electrode were most likely to be activated, especially at low-stimulation amplitudes (Fig. 2*D4*).

For model states representing neuropathic pain, we randomly varied the values of several parameters related to disinhibition in the network. First, we reduced the GABAergic conductance by up to 50% of the initial value (Moore et al., 2002). Second, the reversal potentials of inhibitory synapses were shifted in 4-mV increments by up to 16 mV (Coull et al., 2003). Third, the number of Aδ and C fibers that were active was increased by up to 50% in surround nodes to represent an expanded pain area. Finally, the conductance of Aβ fiber inputs to inhibitory interneurons was reduced by up to 50% (Zhang et al., 2014). Each of these changes was implemented separately for each node, and the degree of disinhibition from each model variant was selected using Latin hypercube sampling with 30 different simulations.

### Animal preparation

Recordings from DC axons and DH neurons were conducted in separate experiments on male Sprague Dawley rats (300–500 g). DH neuron responses were recorded from eight rats and DC responses were recorded from six rats. All animal care and experimental procedures were approved by the Institutional Animal Care and Use Committee at Duke University. Rats were housed in pairs and were initially anesthetized with isoflurane (3.0%, inhaled, Abbott Laboratories) and then urethane (1.2 g/kg, s.c.; Sigma-Aldrich). Anesthesia was supplemented with a second dose of urethane (0.4 g/kg, i.p.) and a third dose (0.1 g/kg, i.p.) if pinching the hindpaw evoked a withdrawal reflex. A tracheotomy was performed to allow intubation to maintain respiration during paralysis. Respiration, heart rate and SpO_2_ were monitored throughout the surgical procedure (PhysioSuite; Kent Scientific). Temperature was monitored using a rectal probe and maintained between 35°C and 37°C using a heating blanket (Gaymar T/Pump). Rats were attached to a stereotaxic frame using ear bars and vertebral clamps attached to T12 and L2 (Kopf instruments). An incision was made over the left hindlimb to expose the sciatic nerve and its distal branches. A laminectomy was performed to expose the spinal cord between T13 and L1. For DC recordings, a second laminectomy was performed to expose the cervical (C6) segment. The dura was resected over the exposed spinal cord segments. Following data collection animals were euthanized with an overdose of Euthasol followed by bilateral thoracotomy.

### DC axon unit recording

**Figure 3.**
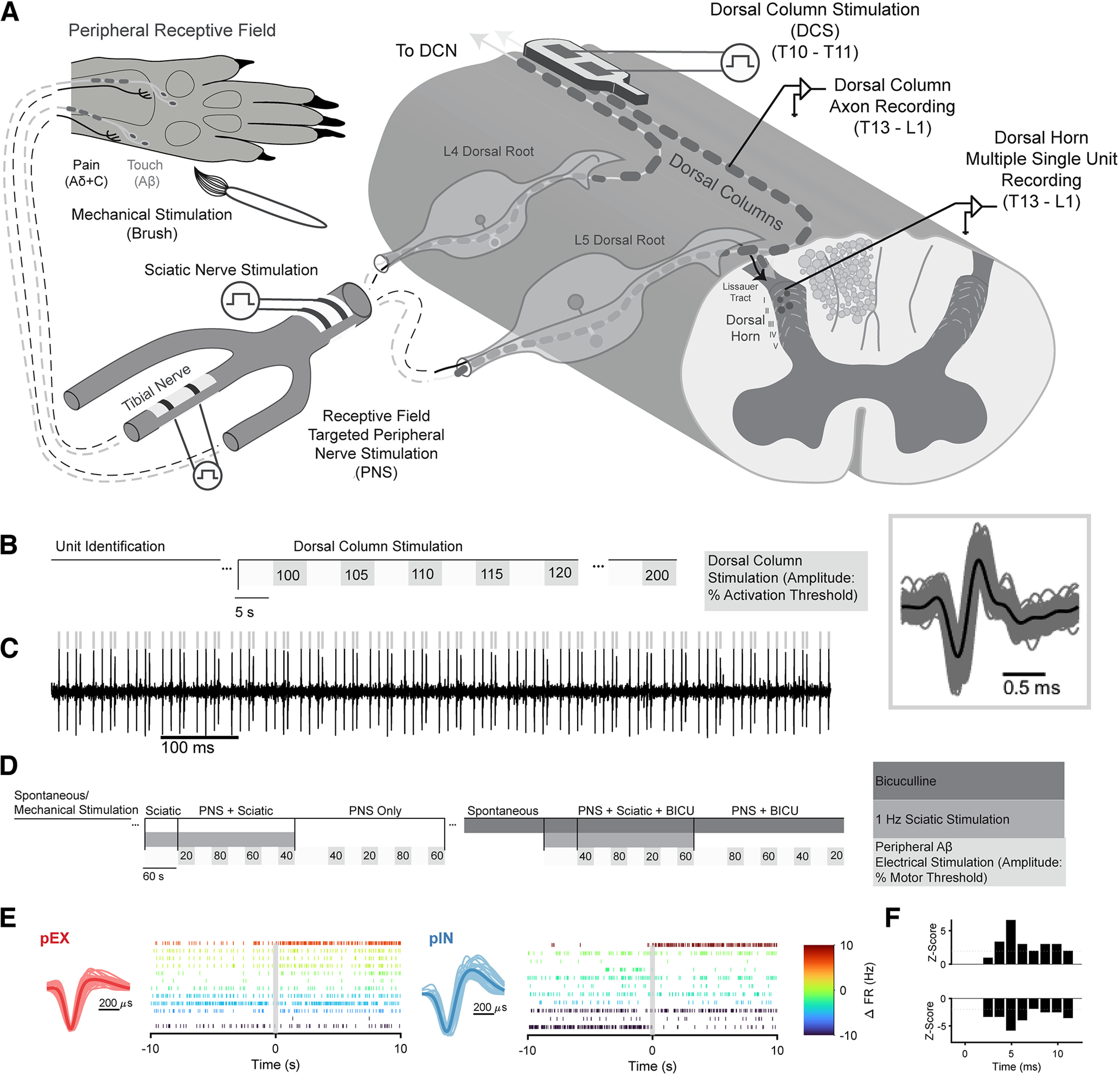
Experiment recording setup. ***A***, Individual DC axons and multiple DH neurons were recorded in separate animals. DC axons were recorded from the lumbar spinal cord using a bipolar tungsten microelectrode. Multiple single units were recorded from the lumbar dorsal horn using a 16- or 32-contact silicon microelectrode. The sciatic nerve was exposed to stimulate individual branches or the entire sciatic nerve. DH neurons were evaluated for their response to receptive field targeted peripheral nerve stimulation and stimulation of the full sciatic nerve. ***B***, Timeline of evaluation of DC axon responses to bipolar stimulation at T10–T11 at different amplitudes. Amplitudes were randomized across trial blocks. ***C***, Example DC axon recording during dorsal column stimulation (DCS). Black trace is raw recording, gray lines indicate detected spikes. The gray box shows the waveforms of the recorded unit. ***D***, Timeline of DH recordings. After identifying units from the receptive field area using mechanical stimulation of the ipsilateral rat hindpaw, spontaneous activity was recorded for at least 10 min. Next, multiple single units were recorded with different (randomized) amplitudes of tibial nerve stimulation with and without concomitant stimulation of the sciatic nerve at C-fiber amplitude. In some animals, neurons were also recorded after applying bicuculline (BICU) intrathecally at the recording site. See Extended Data Figure 3-1 for responses of DH neurons to mechanical stimulation before and after application of BICU. ***E***, Individual units were classified based on their waveform shape (Snyder et al., 2016; Lee et al., 2019). Plots left of raster show the sorted waveforms of individual units and the mean waveform. Raster plots show 10 s of monophasic putatively excitatory (pEX) and biphasic putatively inhibitory (pIN) unit responses to receptive-field targeted stimulation at 60% MT. Raster color represents the change in firing rate over the full 30-s window of stimulation. ***F***, Example Z-scores calculated for one neuron excited by Aβ-ES and one neuron inhibited by Aβ-ES. Neurons were classified as responders if three or more consecutive bins exhibited |z| ≥ 1.96 (Montgomery, 2006).

We recorded responses of 24 individual DC axons from six animals to epidural SCS (Fig. 3*A*) using methods described previously (Crosby et al., 2017). Briefly, DC axons were stimulated using a custom bipolar paddle consisting of two 1.5 × 1 mm platinum contacts spaced 2 mm apart and insulated dorsally using silicone. The electrode was inserted into the epidural space underneath the T10–T11 vertebrae. Motor thresholds (MTs) for DC axon recordings were determined by identifying the lowest amplitude that evoked a visible twitch using 90 Hz/225 μs. Axons were recorded using a bipolar tungsten microelectrode (impedance 8−10 MΩ, tip spacing 190 μm; FHC) inserted into the DC mediolaterally at an angle of 30–70° relative to the sagittal plane. Signals were bandpass filtered from 500 Hz to 5 kHz, amplified by 1000 (XCell3; FHC), further amplified to a total gain of 10,000 (SR560; Stanford Research Systems), and sampled at 20 kHz (PowerLab; ADInstruments). Once a single unit was acquired, it was confirmed to be a single projecting axon using the following criteria: invariant latency to stimulation, faithful response to a short train of 200-Hz stimulation, and the amplitude/waveform matched between activation at the cervical and thoracic locations. The responses of individual DC axons were assessed in response to 5 s of SCS at amplitudes near their individual ATs (Fig. 3*B*). Each stimulation block for DC axon recordings lasted ∼4 min, and units were reconfirmed between stimulation blocks. After identifying units, we filtered the signal and used a custom algorithm (MATLAB R2021a; MathWorks) to find spike times (Fig. 3*C*). The stimulation artifact is ∼0.5 ms wide and occurs ∼1 ms before the start of each action potential; the artifact cannot be distinguished from individual action potentials in Figure 3*C* and was removed from the zoomed in window.

10.1523/ENEURO.0058-22.2022.f3-1 Extended Data Figure 3-1 Differential responses of DH neurons to mechanical stimulation before and after application of bicuculline support separate classification of pEX and pIN neurons. ***A***, Percent of neurons that were responders to different stimulation modalities in recordings without bicuculline (non-BICU) and following application of bicuculline (BICU). Responders indicate neurons that exhibited a significant change in activity quantified by their PSTH for poststimulation compared to prestimulation. Neurons are split up by pEX or pIN waveform shape. ***B***, Change in firing rate for spontaneous activity, brush responses, and crush responses following application of bicuculline. Colors represent the change from the non-BICU response to the BICU response. ***C***, Mean change in firing rates across all neurons. Error bars are SE. Grey asterisks represent a significant change in responses (*t* test, *p < 0.05, **p < 0.01, ***p < 0.001). Black asterisks represent significant differences between pEX and pIN neurons (kstest2, *p < 0.05, **p < 0.01). Download Figure 3-1, TIF file.

### DH unit recording, classification, and sorting

We recorded responses of 215 DH neurons from eight rats to Aβ-ES (Fig. 3*A*). Although the dorsal columns follow a somatotopic organization based on fiber entry level (Smith and Bennett, 1987), precisely targeting groups of dorsal column fibers in a rodent spinal cord is difficult, as rodents cannot directly indicate where sensations are generated by SCS. However, since peripheral nerves correspond to well-defined receptive field areas in accordance with the somatotopic organization (Swett and Woolf, 1985; Kambiz et al., 2014), for *in vivo* DH neuron recordings, we used peripheral Aβ-ES rather than dorsal column stimulation to stimulate selectively center and surround receptive field areas. For DH recording experiments, MTs were measured by stimulating the tibial branch of the sciatic nerve using a bipolar cuff electrode with a 0.5- to 1.0-mm internal diameter (Microprobes for Life Science) with 50-Hz/200-μs stimuli in all animals. MTs were also measured with 90-Hz/225-μs and/or 50-Hz/300-μs stimuli in four animals, and MTs were not significantly different between parameters. Following MT testing, rats were paralyzed with gallamine triethiodide (1 ml/h at 0.2 g/ml) injected through an intraperitoneal catheter. To record single units, 16- or 32-contact recording electrodes (NeuroNexus) were slowly inserted into the lumbar spinal cord exposed by the T13-L1 laminectomy; 16-contact electrodes had a recording span of 375 μm (Neuronexus electrode A1x16-Poly2-5mm-50s-177-A16) and 32-contact electrodes had a recording span of 275 μm (Neuronexus electrode A1x32-Poly3-5mm-25s-177). Electrodes were inserted at a 30–45° rostral-caudal and medial-lateral angle just medial to the dorsal root entry zone. Electrode depths spanned from ∼50 to 800 μm with an average depth of 400 μm. The rostral-caudal position of each electrode was identified with respect to the vertebral level. Signals were amplified, filtered, and sampled on a 32-channel recording system (MAP System, Plexon). Brushing was used as a search stimulus to identify units within the receptive field. Individual single units were identified online and then confirmed offline and sorted using feature analysis (Offline Sorter V3, Plexon). The primary features used for sorting were the first three principal components, waveform energy and nonlinear energy, and waveform amplitude.

After identifying individual DH units, we quantified responses to 90-Hz/225-μs peripheral electrical stimulation of the tibial or peroneal branch of the sciatic nerve at different stimulation amplitudes (20%, 40%, 60%, and 80% MT; Fig. 3*D*). Responses to peripheral electrical stimulation were also evaluated during ongoing stimulation of the sciatic nerve with a bipolar cuff electrode (Microprobes for Life Science) at 1 Hz and an amplitude 50–60 times MT to drive C-fiber inputs to the DH. In one group of rats (*n* = 2), bicuculline methiodide (Alfa Aesar) was administered following at least one block of baseline recordings (Fig. 3*D*). A total of 10 μl of 0.3 μg/μl bicuculline in 0.9% saline solution was administered intrathecally at the recording site with a Hamilton syringe. A subsequent block of recordings and was initiated after recording spontaneous activity for at least 20 min. Each stimulation block lasted 11 min with ∼20–30 min before each block to record spontaneous activity and responses to mechanical stimulation. Units were confirmed through online sorting before each stimulation block.

We classified individual units by their waveform shape because prior studies found a functional difference between units with monophasic and biphasic waveforms (Lee et al., 2019), and both monophasic and biphasic neurons exhibited a variety of responses to peripheral nerve stimulation (Fig. 3*E*). In prior studies, monophasic neurons were putatively excitatory (pEX) based on their firing patterns and receptive field areas while biphasic neurons were putatively inhibitory (pIN; Lee et al., 2019). Units were classified as pEX, pIN, or unclassified using a custom classification algorithm (MATLAB; MathWorks). The algorithm modeled each average unit waveform as a double exponential (Eq. 1), then extracted key features from the fitted double exponential and its first derivative (Snyder et al., 2016). Single units were classified as pEX or pIN if the confidence that they belonged to the respective class was higher than 60%. We also classified neurons based on responses to mechanical stimulation (brush, press, pinch crush) on the ipsilateral hindpaw. Among neurons classified as pEX that we identified as mechanically responsive, 12% were classified as high threshold (only responded to crush), 76% were wide-dynamic range neurons (increasing response rates with increasing stimulus strength), and 12% were low-threshold (only responded to brush and/or press). Among neurons classified as pIN that were mechanically responsive, 18% were high threshold, 55% were wide-dynamic range, and 27% were low-threshold:

(1) $$V=a_{1}*exp\left(-{\left(\frac{x-b_{1}}{c_{1}}\right)}^{2}\right)+a_{2}*exp\left(-{\left(\frac{x-b_{2}}{c_{2}}\right)}^{2}\right).$$

We constructed peristimulus time histograms (PSTHs) of neural activity to identify neurons that were responsive to stimulation (Fig. 3*F*). We expected that application of bicuculline would differentially affect pEX and pIN neuron responses to mechanical stimulation. Bicuculline substantially increased the percentage of pEX neurons that responded to mechanical brush and crush inputs (from 40% to 70% for brush and from 43% to 50% for crush) but did not change the response rate of pIN neurons that responded to mechanical inputs (Extended Data Fig. 3-1). However, bicuculline unmasked a significant change in brush responses for both pEX and pIN neurons (*p* < 0.001; Extended Data Fig. 3-1), but the change in brush responses were significantly larger in pEX neurons [two-sample Kolmogorov–Smirnov test (kstest2), *p* < 0.05]. Bicuculline also unmasked a significant change in responses to crush inputs in both neuron classes (*t* test, *p* < 0.001), but the changes did not differ between pEX and pIN neurons. Further, while application of bicuculline increased the activity of neurons both in the presence and absence of mechanical stimulation, pIN neurons showed a significant increase in spontaneous activity compared with pEX neurons (kstest2, *p* < 0.01). Taken together, these differential effects of bicuculline on AP morphology-classified pEX and pIN neurons support our waveform-based classification and justify independently considering the effects of Aβ-ES separately on each subclass of neuron.

We compared single unit activity during SCS to spontaneous activity before stimulation to determine responders to stimulation following established criteria (Zhang et al., 2015). First, we objectively determined the optimal bin width for each neuron and each stimulation frequency by evaluating the spike count per bin at each frequency (Shimazaki and Shinomoto, 2007), and we compared activity during SCS to spontaneous activity before stimulation with the same bin widths. We identified bins with the stimulation artifact present in the stimulation condition according to the bin width, and to enable a direct bin-to-bin comparison, we omitted activity in these corresponding bins from spontaneous activity. Neurons were classified as responders if stimulation activity compared with baseline spontaneous activity was significantly different (z > 1.96) in three or more consecutive bins. The responses of all neurons are shown, but neurons had to have a firing rate of at least 1.5 Hz and respond to at least one amplitude to be included in the clustering analyses.

Individual neuron responses were normalized to their greatest change in firing rate at each amplitude such that the largest change corresponded to +1 for excitatory responses or −1 for inhibitory responses (Lemay and Grill, 2004). We clustered normalized neurons responses using fuzzy c-means clustering (m = 2.0, max iterations = 500, minimum improvement = 1e-5) based on the first two principal components of their response to all amplitudes. We identified the major cluster groups with between two and six clusters and used the silhouette evaluation or Davies–Bouldin evaluation to determine the optimal number of clusters for each group (Davies and Bouldin, 1979; Rousseeuw, 1987).

## Results

We quantified the effect of Aβ electrical stimulation on the activity of DC axons and DH neurons in computational models and *in vivo* experiments. We initially used stimulation parameters (90 Hz, 225 μs/phase) consistent with those of clinically effective low-frequency subperception SCS, 90-Hz SCS at amplitudes just above the activation threshold for individual DC axons drove asynchronous firing in both model DC axons and individual DC axons recorded *in vivo*. When applied as the Aβ fiber inputs to a computational model of the DH network, these irregular spiking patterns led to greater suppression of model WDR neurons than did regular spiking patterns from DC axon responses evoked by higher stimulation amplitudes. We quantified the effects of electrical stimulation on DH neurons *in vivo* across functional classes (putatively excitatory or inhibitory neurons), recording location, and stimulation parameters. Since identifying receptive fields using SCS via pain-paresthesia overlap mapping is impractical in rodents, we used peripheral Aβ-ES, rather than DC stimulation, to stimulate selectively center and surround receptive field areas. Stimulation parameters and recording location both had strong effects on neural responses, but neuron functional class did not have a significant effect. Importantly, data from both the DH model and *in vivo* experiments indicated that the rostral-caudal location, rather than the identity of the neural targets, was responsible for generating substantial intersegmental inhibition in the spinal dorsal horn. In a subset of animals, we applied the GABA_A_ receptor antagonist bicuculline to represent the loss of inhibition that occurs in chronic pain states (Moore et al., 2002; Braz et al., 2012). Disinhibition following bicuculline unmasked activity in putatively excitatory neurons and revealed differences in neuron responses to Aβ-ES, further supporting a spinal GABAergic inhibitory mechanism mediating the effects of stimulation. Finally, we compared model network responses to different SCS paradigms in network states both before and after impairing inhibition, and from these results, proposed possible methods to optimize stimulation parameters, even following GABAergic disinhibition, by exploiting surround inhibition.

### Low-amplitude SCS drove irregular spiking in DC axons

**Figure 4.**
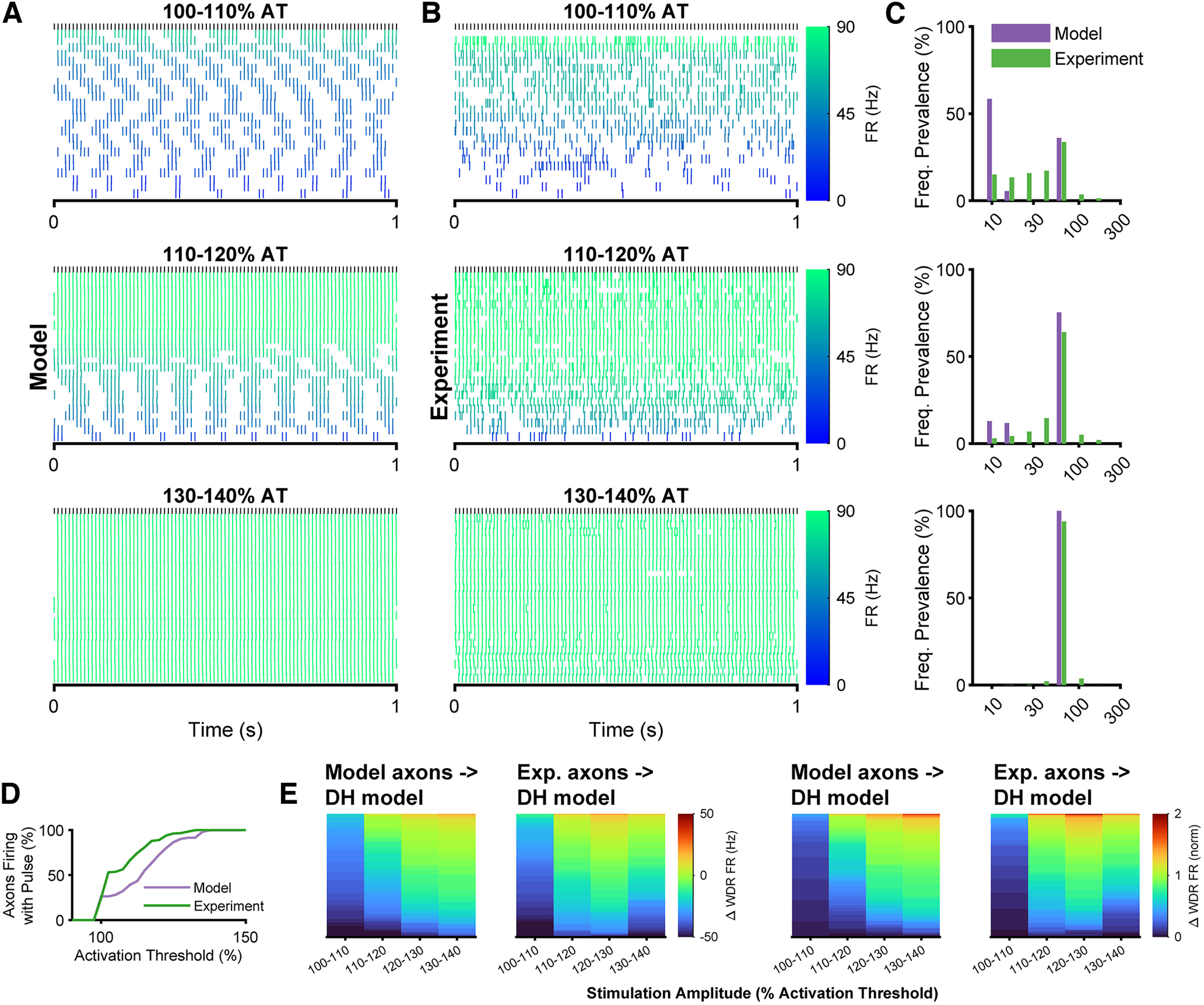
DC axon responses to dorsal column stimulation. ***A***, Raster plots of individual model axon responses to 90-Hz/225-μs stimulation at different amplitudes, normalized to each axon’s activation threshold (AT) and divided into three amplitude subgroups. The color of the raster indicates the average firing rate of each axon. ***B***, Raster plots of 24 individual DC axon responses recorded *in vivo* to 90-Hz/225-μs stimulation at amplitudes normalized to the AT of each axon. ***C***, Prevalence of firing frequencies across the population of DC axons for model and experiment. Frequency prevalence was calculated from the interspike interval (ISI) probability normalized to the average stimulation frequency of each bin. ***D***, Percent of axons that responded to an individual stimulation pulse as a function of stimulation amplitude for the model DC axons and experimental recordings. ***E***, Model WDR responses to DC axon inputs across 100 trials at each amplitude. Raw changes in WDR firing rate (left) and change in WDR firing rate normalized to the baseline firing rate (right) for both model and experimental DC spike times.

We quantified the response of DC axons to different amplitudes of 90-Hz SCS. The spiking patterns did not always match the stimulation patterns, and both model axons (Fig. 4*A*) and axons recorded *in vivo* (Fig. 4*B*) exhibited bursting when stimulated at 100–140% of activation threshold (AT). Although the experimental responses showed greater variability in spiking patterns, both model and experimental axons exhibited bursts dominated by 90-Hz spiking that increased in length with stimulation amplitude (Fig. 4*C*), and immediate entrainment to 90 Hz at 100% AT was never observed in either model or experiment. Furthermore, both model and experimental axons exhibited spiking asynchronous with the stimulation pulses at low-stimulation amplitudes (Fig. 4*D*), and only 25% of axons fired reliably in response to any single specific stimulation pulse at 100% of their AT. However, the majority (72% of model and 86% of experimental) of axons began firing synchronously with the stimulation pulses as stimulation amplitude was increased to ≥120% AT. Thus, DC axons exhibited variable patterns of activation strongly dependent on the amplitude of stimulation, and, in particular, low amplitude stimulation evoked asynchronous bursting activity.

### Irregular spiking in DC axons drove inhibition in dorsal horn network model

Both model-calculated and experimentally recorded patterns of DC axon activity were applied as the Aβ fiber inputs to the biophysically based DH network model (Fig. 2), and changes in model WDR neuron activity were quantified. Interestingly, inhibition of model WDR neurons was greatest by model-generated DC axon spiking patterns evoked at the lowest amplitude (100–110% AT), and patterns from higher amplitudes resulted in less inhibition of WDR neurons (Fig. 4*E*). Similarly, with DC axon inputs from *in vivo* recordings, the greatest inhibition of model WDR neurons also occurred with patterns evoked at lower stimulation amplitudes, and the average reduction in WDR model neuron spiking was similar by patterns taken from model axons and from *in vivo* recordings across stimulation amplitudes. Model WDR neurons also received excitatory inputs from Aβ fibers (Fig. 2*A1*), and the excitatory effect of higher stimulation amplitudes was because of increased direct excitation of WDR neurons. In contrast to effects on model WDR neurons by model-derived patterns of DC axon activity, model WDR neurons were more inhibited by patterns of DC axon activity derived from *in vivo* recordings during stimulation at 130–140% AT than at 120–130% AT. Nevertheless, the model predicted that irregular spiking inputs from DC axons led to stronger net inhibition of excitatory neurons in the DH network than regular spiking inputs from higher amplitude stimulation. Collectively, this indicated that both the pattern of DC axon activity and the relative balance of direct excitation from Aβ fibers and indirect (interneuron-mediated) inhibition altered WDR neuron activity.

### Inhibition of model WDR neuron activity was maximized by engaging surround inhibition and selecting specific SCS parameters

**Figure 5.**
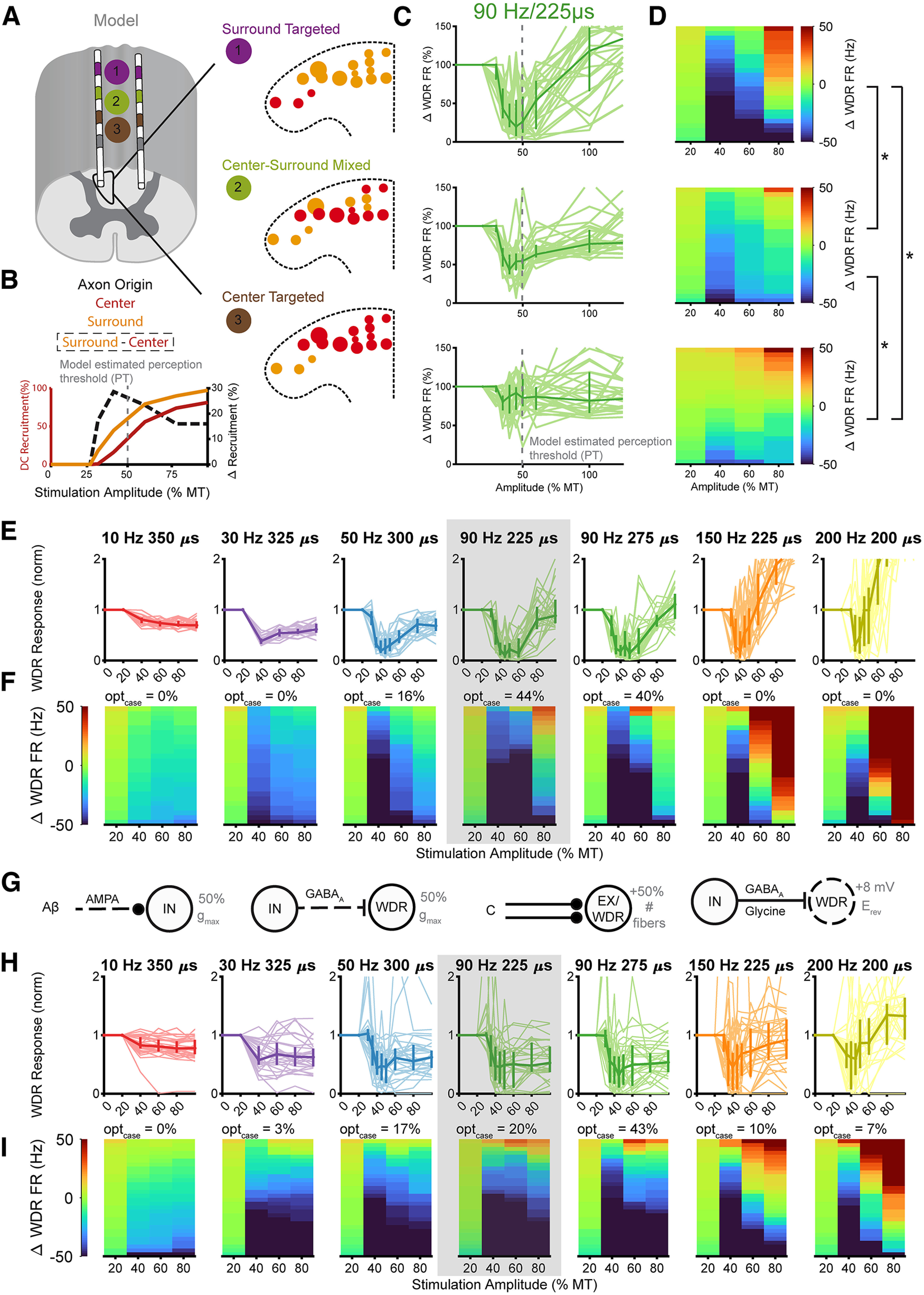
Model responses to low-rate, low-amplitude SCS depend on spatial targeting, stimulation parameters (amplitude, pulse width, rate), and pain state. ***A***, Spatial targeting in the model was simulated by altering the population of DC axons that was activated at each amplitude. We changed the peripheral origin of model DC axons based on the rostral-caudal position of stimulation. For stimulation in the most caudal position (brown), axons from the center of the peripheral receptive field were positioned in the most medial and dorsal positions within the DCs (see Fig. 1; Smith and Bennett, 1987). Conversely, for stimulation in the most rostral position (purple), axons from the surround were in the most medial and dorsal positions. Targeting in between (green) mixed center and surround axons. ***B***, Change in DC axon recruitment across stimulation amplitudes with surround targeting (purple). Surround and center axon recruitment was best differentiated at 40–50% MT, below the estimated PT. ***C***, Responses of model WDR neurons with each stimulation target, i.e., complement of DC axons that were activated. Vertical dashed line indicates model PT estimated as 50% of the MT (Shechter et al., 2013). ***D***, Raw change in WDR firing rate at 20%, 40%, 60%, and 80% of estimated MT. Lines with asterisks (*) represent significant changes in the population response between stimulation positions (ANOVA, *post hoc* Tukey’s test, *p* < 0.05). ***E***, Model WDR neuron responses to different amplitudes of SCS at seven different frequency/pulse width combinations. Each line represents one individual distribution of DC axon activation. Bold lines represent median response and error bars represent the 25th and 75th percentiles of the responses. ***F***, Raw changes in firing rate at 20%, 40%, 60%, and 80% of model estimated MT. Estimated model MT was 100 μA, so 20% MT was 20-μA stimulation. Responses are sorted by the change in firing rate at each amplitude. ***G***, Representation of modeled changes in network states representing neuropathic pain: reduction in conductance of Aβ fiber weight to the inhibitory interneuron, reduction in the GABAergic conductance from the inhibitory interneuron to WDR projection neuron, increase in the number of active C/Aδ fibers, and increase in the reversal potential of inhibitory synapses. ***H***, Model WDR neuron responses by stimulation amplitude at seven different combinations of frequency and pulse width. Light lines represent individual responses from 30 different model pain states. Dark lines represent the median response and error bars represent the 25th and 75th percentiles. ***I***, Sorted raw changes in firing rate for all model WDR neurons at each combination of frequency and pulse width.

The prior simulations predicted the effect of stimulation amplitude on DC activity, and we also quantified how the population of activated DC axons altered responses of DH neurons by altering the balance of direct excitation and indirect (interneuron-mediated) inhibition of WDR neurons. Surround inhibition in the DH is hypothesized to depend on precise topography of Aβ fiber stimulation (Hillman and Wall, 1969; Lee et al., 2019; Fan and Sdrulla, 2020) and involve segmental GABAergic mechanisms (Zhang et al., 2014, 2015). Therefore, we compared changes in model WDR neuron activity evoked by stimulation delivered to different receptive field areas by altering the proportion of center versus surround model DC fibers that were activated (Fig. 5*A*). This shift in the proportion of activated center versus surround fibers is analogous to shifting the contacts that are active during SCS to target different fiber groups, as fibers from relative surround receptive field areas are positioned more dorsally and medially in more rostral positions along the cord (Smith and Bennett, 1987; Niu et al., 2013). In general, stimulation suppressed the activity of model WDR neurons relative to model WDR neuron activity during a modeled pain input, but model WDR neuron responses were dependent on the origin of activated DC axons (ANOVA, *p* < 0.05, *post hoc* Tukey’s test, *p* < 0.05), i.e., the proportion of activated DC axons that arose from the surround receptive field area versus the center receptive field area. Activating DC fibers from the surround receptive field was necessary for maximal suppression of the model WDR neuron at lower stimulation amplitudes (Fig. 5*B*). Maximizing WDR suppression at a putatively subperception amplitude (40% MT) was achieved when we assumed SCS activated axons that originated primarily from the surround receptive field (Fig. 5*C*,*D*). Furthermore, model WDR suppression exhibited a nonmonotonic relationship with stimulation amplitude, which is explained by the shift from indirect (interneuron-mediated) inhibition to direct activation as stimulation amplitude was increased and more center model DC axons originating from the center were activated. Finally, the effects on WDR activity were rapid. Consistent with clinical observations, models predicted that inhibition of WDR neuron activity occurred within seconds of the start of stimulation in response to low rate but not high rate SCS (Extended Data Fig. 2-1*A*).

To identify the optimal parameter settings for fast-acting subperception SCS in the computational model, we quantified the effects of a broad range of stimulation frequencies and pulse widths (selected to deliver a similar neural dose by adjusting both the frequency and pulse width of stimulation; Paz-Solís et al., 2022) on inhibition of model WDR neurons (Fig. 5*E*). Increasing the frequency of SCS targeted to surround receptive fields increased the maximum reduction in WDR firing rate but decreased the range of amplitudes that produced a reduction in WDR firing rate. For example, the range of stimulation amplitudes that reduced the firing rate of model WDR neurons by at least 50% was 35−60% of MT for 50-Hz/300-μs stimulation, but only 35−40% for 200-Hz/200-μs stimulation. Higher stimulation frequencies also excited WDR neurons at higher amplitudes of SCS (Fig. 5*F*). For each of the 25 random selections of DC axons (Fig. 2*D*), we determined the stimulation parameters that produced the maximum reduction in WDR firing rate for amplitudes between 30% and 60% of MT (amplitudes within the therapeutic range). 50 Hz/300 μs produced the greatest inhibition in 16% (4/25) cases, 90 Hz/225 μs was optimal in 44% cases, and 90 Hz/275 μs was optimal in the remaining 40% cases. The fact that 90-Hz stimulation was optimal in most model conditions and the use of 90-Hz stimulation during the clinical study using fast-acting subperception SCS (Metzger et al., 2021) justified the use of 90-Hz stimulation for our *in vivo* experiments.

We also simulated changes in model WDR activity after multiple impairments to inhibition to represent changes observed with chronic pain progression (see Materials and Methods; Fig. 5*G*). These simulations of diverse pain states exhibited more variability in the model WDR responses to stimulation than the healthy model simulations but showed similar trends (Fig. 5*H*). Following pain state induction, 90 Hz/275 μs was the most effective parameter setting in 43% (13/30) of pain states, and parameter settings from 30 to 200 Hz were optimal for at least one pain state (Fig. 5*I*). Nonetheless, these results still indicated that 90-Hz stimulation was optimal for our *in vivo* experiments, even with disrupted dorsal horn inhibitory mechanisms.

### Inhibition of DH neurons by low-amplitude Aβ-ES was disrupted by bicuculline

**Figure 6.**
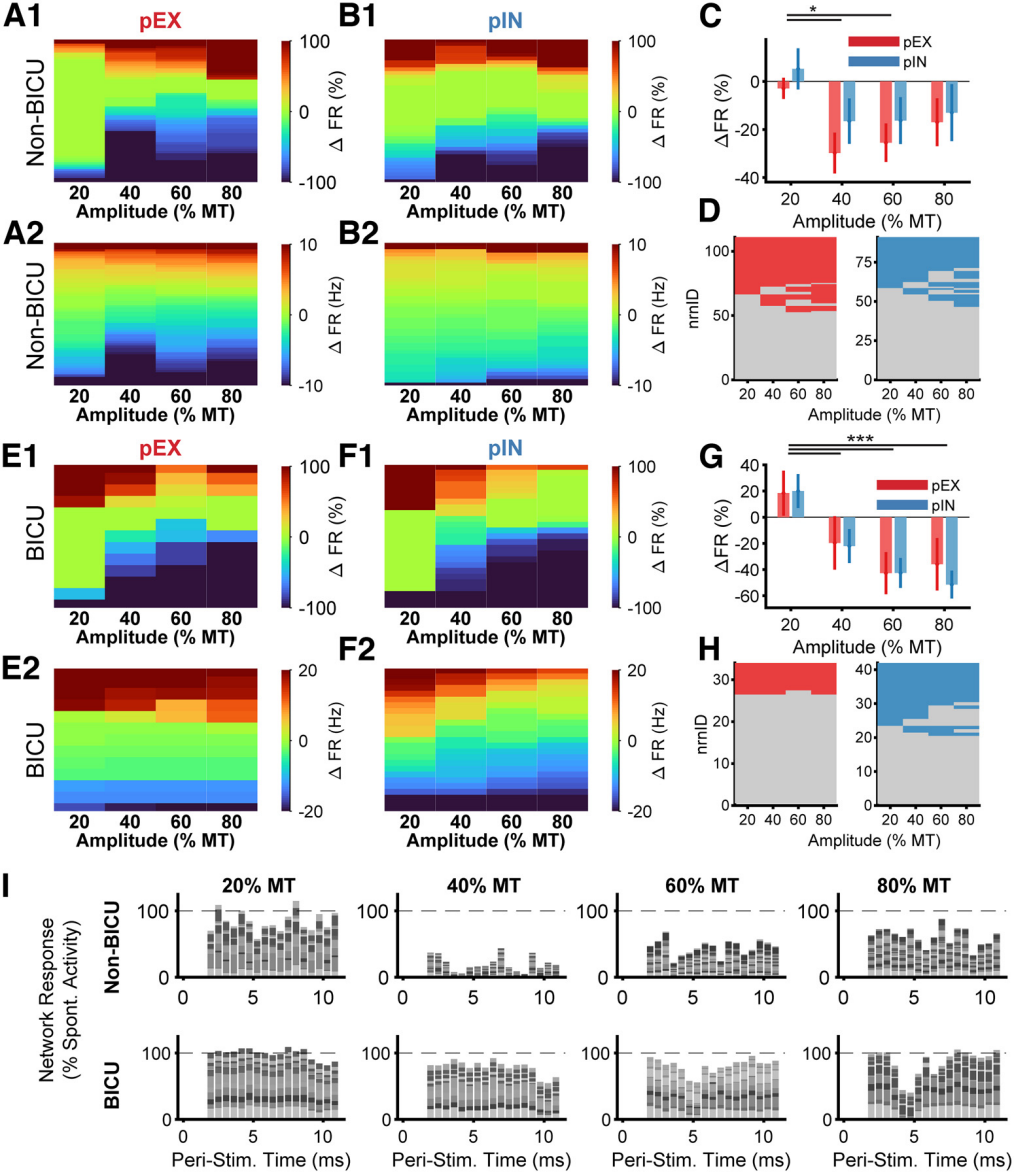
Segmental application of bicuculline disrupted inhibition from Aβ-ES. ***A***, Changes in firing rate compared with spontaneous activity of pEX neurons before application of bicuculline during 90-Hz stimulation at different stimulation amplitudes (percentage of motor threshold). ***A1***, Changes in firing rate normalized to the peak change in firing rate. ***A2***, Raw changes in firing rate. ***B***, Same as ***A***, but for pIN neurons. ***C***, Mean normalized changes in pEX and pIN neuron responses to stimulation at different amplitudes. ***D***, pEX (left) and pIN (right) neurons counted as responders for each stimulation amplitude. Colored boxes indicate neurons that are responders to stimulation and gray boxes indicate nonresponders. See Extended Data Figure 6-1 for responses to low-amplitude Aβ-ES and C-fiber sciatic nerve stimulation. ***E–H***, Same as ***A–D***, but for neurons recorded after application of bicuculline. Error bars represent SE. Asterisks indicate significant difference between stimulation amplitudes (rmANOVA, *p* < 0.05, *post hoc* Tukey’s test, *p < 0.05, **p < 0.01, ***p < 0.001). ***I***, Example PSTHs of simultaneously recorded units at each amplitude and for nonbicuculline and bicuculline conditions. Neurons were normalized individually by dividing by their spontaneous firing rate (not across amplitudes as in ***B***, ***F***). Includes both responders and nonresponders, but neurons with large excitatory responses (>200% of spontaneous firing rate) were not included.

We recorded responses of multiple single units in the DH of anesthetized rats to quantify the effects of stimulation location and stimulation parameters on neural activity. The effects of stimulation on DH neurons were heterogeneous (Zhang et al., 2015), and we classified neurons into two functional classes: putatively excitatory (pEX) or putatively inhibitory (pIN) according to the shape of the recorded AP (see Materials and Methods; Lee et al., 2019). We used peripheral Aβ-ES rather than DC Aβ-ES to study the effects of targeting stimulation to specific receptive field areas. We focused on short-term effects of stimulation, and quantified changes in neural activity over a 30-s window following stimulation onset. We plotted the changes in activity of all neurons during stimulation and detected no differences in the distribution of inhibitory versus excitatory neuron responses to Aβ-ES between the pEX and pIN neuron classes (Fig. 6*A*,*B*, ANOVA, *p* = 0.9). The responses of both pEX and pIN were dependent on stimulation amplitude, and consistent with the effects on model neurons, stimulation at 40 and 60% MT significantly reduced pEX and pIN activity compared with 20% MT for both normalized and raw responses (Fig. 6*C*, rmANOVA, *p* < 0.001, *post hoc* Tukey’s test, *p* < 0.001). Similarly, there was a significant effect of stimulation amplitude (rmANOVA, *p* < 0.001), but not neuron class (*p* = 0.57) on DH neuron responses to Aβ-ES delivered during coincident high-amplitude (above C-fiber threshold) sciatic nerve stimulation that increased the mean firing rate of recorded neurons before application of Aβ-ES (Extended Data Fig. 6-1). Taken together, these results are the first to demonstrate that putatively subperception (40% MT or below; Crosby et al., 2017) Aβ-ES is sufficient to produce effects in DH neurons and corroborate our hypothesis that low-level Aβ-fiber activation drives these effects.

10.1523/ENEURO.0058-22.2022.f6-1 Extended Data Figure 6-1 Recorded neural responses to low-amplitude Aβ-ES at 90-Hz and 225-μs combined sciatic nerve stimulation at amplitude to activate C-fibers. ***A***, Responses of pEX neurons in animals without bicuculline showing changes in firing rate normalized to the peak change in firing rate (***A1***) and raw changes in firing rate (***A2***). ***B***, Same as ***A***, but for pIN neurons. ***C***, Mean normalized changes in pEX and pIN neuron responses to stimulation at different amplitudes. ***D***, pEX (left) and pIN (right) neurons counted as responders for each stimulation amplitude. Colored boxes indicate neurons that are responders to stimulation. Error bars represent SE. Asterisks indicate significant difference between stimulation amplitudes (rmANOVA, *p* < 0.05, *post hoc* Tukey’s test, *p < 0.05, **p < 0.01, ***p < 0.001). Download Figure 6-1, TIF file.

Aβ-ES also reduced the activity of pEX and pIN neurons following application of bicuculline, but, unlike prebicuculline recordings, inhibition of pEX and pIN activity increased monotonically with stimulation amplitude (Fig. 6*E–H*). The activity of both pEX and pIN neurons was reduced more by Aβ-ES at 40%, 60%, and 80% MT compared with 20% MT (Fig. 6*G*, rmANOVA, *post hoc* Tukey’s test, *p* < 0.001), and again there was no effect of neuron class (*p* = 0.77). There was a shift from strong and persistent inhibition by Aβ-ES at 40% MT to weaker and more transient inhibition by Aβ-ES at 80% MT as seen in the PSTHs of individual neurons that were recorded simultaneously (Fig. 6*I*). Interestingly, the proportion of pEX but not pIN neurons responding to Aβ-ES (i.e., exhibiting a significant change in activity poststimulation compared with prestimulation) was substantially reduced by application of bicuculline compared with control (nonbicuculline) recordings. This supports a segmentally mediated but complex GABAergic mechanism as a driver for inhibition mediated by low-amplitude Aβ-ES (pEX: 53% responders to stimulation in control recordings vs 24% in bicuculline recordings, pIN: 49% control vs 52% bicuculline; Extended Data Fig. 3-1). However, the fact that stimulation remained effective despite reduced GABAergic inhibition suggests that high-amplitude Aβ-ES can recruit additional inhibitory mechanisms, such as glycinergic inhibition.

### Engaging surround inhibition maximized inhibition of DH neurons

**Figure 7.**
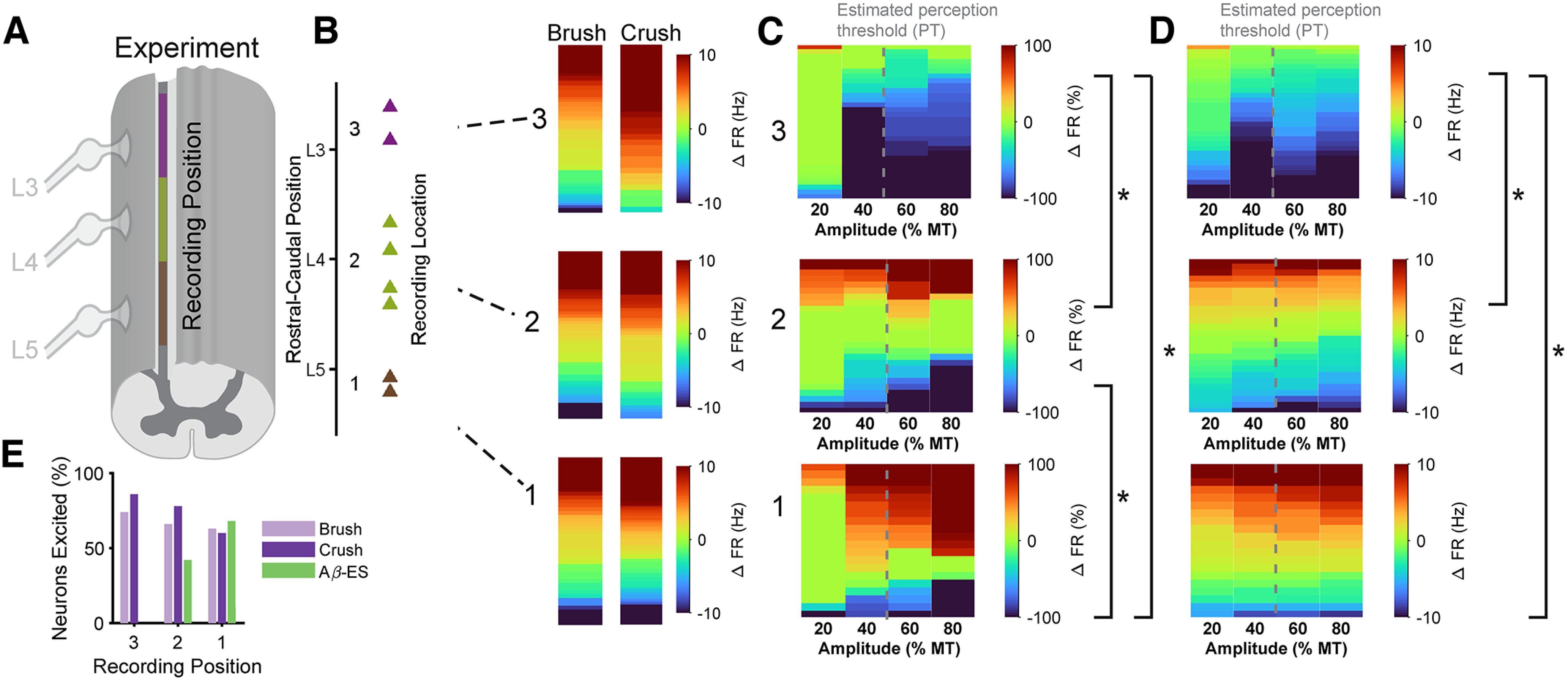
Responses of DH neurons to Aβ-ES depend on stimulation amplitude and location (receptive field targeting). ***A***, Individual DH neuron responses were sorted into three groups based on the location where they were recorded. ***B***, Normalized changes in pEX neuron activity divided by recording position. pIN neuron activity did not depend on recording position (Extended Data Fig. 7-1). ***C***, False color maps of changes in pEX neuron activity versus baseline activity split by recording position. ***D***, Same as ***C***, but raw firing rate changes versus baseline. Lines with asterisks (*) represent significant changes in the population response between stimulation positions (ANOVA, post hoc Tukey’s test, *p*,0.05). In ***C***, ***D***, data are from pEX neurons classified as responders by recording location, and the gray dotted lines between 40% an 60% MT represents estimated PT. ***E***, Percent of pEX neurons excited compared with spontaneous activity at each recording position for peripheral mechanical stimuli (brush, crush) and for Aβ-ES.

Dorsal horn neurons are roughly arranged rostrocaudally by receptive field (Swett and Woolf, 1985). We segregated the individual *in vivo* recordings of pEX dorsal horn neurons by their rostrocaudal location along the spinal cord (Fig. 7*A*,*B*) while stimulating the same peripheral receptive field. In this way, the same peripheral inputs represented either center or surround receptive field inputs to different neurons, depending on their rostrocaudal locations, and this also enabled comparison to model neuron responses across relative positions. Mechanical brush and crush inputs were delivered on the plantar surface of the hind paw, the center receptive field area of the tibial nerve (Swett and Woolf, 1985; Kambiz et al., 2014). Since we stimulated peripherally the tibial branch of the sciatic nerve, most stimulation-affected afferents should enter through the L5 and L4 spinal roots (Swett et al., 1991) corresponding to the “center” receptive field area and recording positions 1 and 2, respectively. Position three corresponded to the L3 spinal root, rostral to the primary entry level of afferents from the tibial nerve and therefore likely corresponding to a “surround” receptive field.

10.1523/ENEURO.0058-22.2022.f7-1 Extended Data Figure 7-1 DH responses to Aβ-ES for pIN neurons do not depend on receptive field targeting. ***A***) Individual DH neuron responses were sorted into three groups based on the location where they were recorded. ***B***) Normalized changes in pIN neuron activity divided by recording position. ***C***) False color maps of changes in pIN neuron activity vs. baseline activity split by recording position. ***D***) Same as ***C***, but raw firing rate changes vs. baseline. In ***C*** and ***D***, data are from pIN neurons classified as responders by recording location, and the gray dotted lines between 40% an 60% MT represents estimated PT. Download Figure 7-1, TIF file.

Neural responses were dependent on recording position (Fig. 7; ANOVA, *p* < 0.001) and were different between position three versus both positions 1 and 2 (*post hoc* Tukey’s test, *p* < 0.05). However, responses for pIN neurons were not dependent on position (ANOVA, *p* = 0.15; Extended Data Fig. 7-1). Consistent with model results, responses of neurons in position 1 were mostly excitatory while responses in position 2 showed a mix of excitation and inhibition, and responses in position three were strongly inhibitory (Fig. 7*C*,*D*). Thus, the position-dependent responses *in vivo* are consistent with the center-surround architecture represented in the model, with each zone approximately corresponding to responses in each recording position (Hillman and Wall, 1969). We only studied the effects of stimulation over 30-s windows *in vivo*, but as in the model, the changes in firing rates typically occurred within seconds of the onset of stimulation and persisted for the entire stimulation window (Extended Data Fig. 2-1).

The center-surround architecture was also examined by considering the proportion of pEX neurons excited at each recording location (Fig. 7*E*). The majority of pEX neurons in position 1 were excited by Aβ-ES, while all pEX neurons in position three were inhibited by Aβ-ES. In contrast, the percentage of pEX neurons excited by both brush and crush stimulation increased from position three to position 1, indicating that inhibition from peripheral Aβ-ES was distinct from the primary area of excitation from mechanical stimulation. These spatial relationships corroborate the center excitation-surround inhibition spatial organization of the model and support a need for precise spatial targeting of subperception Aβ-ES to maximize efficacy.

### Response clusters affected differently by 50- and 90-Hz peripheral Aβ-ES after disinhibition

**Figure 8.**
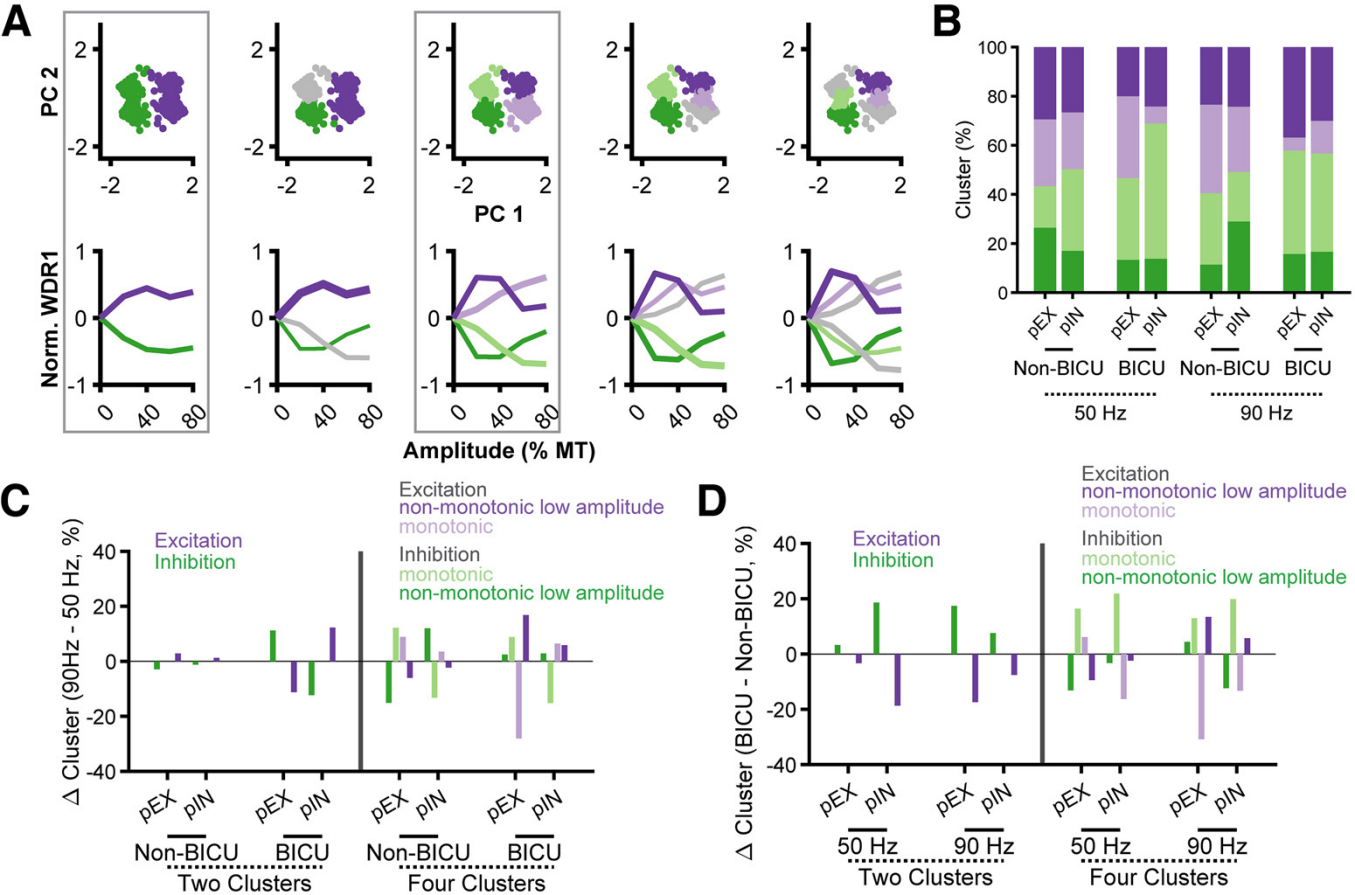
Bicuculline (BICU) affects clustered network responses to stimulation. ***A***, Normalized neuron responses to peripheral Aβ-ES at 50 Hz and 90 Hz were clustered from their first two principal components using fuzzy c-means clustering into two to five clusters. Top plots show responses in component space. Bottom plots show the mean normalized response of each cluster by amplitude. Quantitative analysis identified either two (Davies and Bouldin, 1979) or four (Rousseeuw, 1987) as the optimal number of clusters, and boxes were placed around these plots. ***B***, Percent of neurons in each cluster split up by neuron class (pEX and pIN), pain condition (non-BICU and BICU), and stimulation type (50- and 90-Hz Aβ-ES). Color corresponds to the group with four clusters in ***A***. ***C***, Change in percentage of neurons in each cluster between 90- and 50-Hz Aβ-ES. Responses are grouped by pain condition and color corresponds to the clusters in ***A*** with two or four clusters. ***D***, Change in percentage of neurons in each cluster after application of bicuculline. Responses are grouped by stimulation frequency, and colors are the same as ***C***.

We categorized individual neurons by clustering the normalized responses to stimulation at different amplitudes. Fuzzy c-means clustering of the principal components of each response identified heterogeneous response types across the population of recorded neurons. Responses were optimally divided into two clusters (Davies–Bouldin metric; Davies and Bouldin, 1979) or four clusters (silhouette metric; Rousseeuw, 1987; Fig. 8*A*, boxes), and each line represents the average response of each neuron within that cluster for different stimulation amplitudes (Fig. 8*A*). Using two clusters divided responses into “excited” (purple) or “inhibited” (green) responses. Neurons within the “excited” cluster increased net excitation in response to stronger stimulation amplitudes, and “inhibited” responses exhibited the inverse behavior. Using four clusters divided responses into “monotonic excited” (light purple), “low-amplitude excited” (dark purple), “low-amplitude inhibited” (dark green), and “monotonic inhibited” responses (Fig. 8*A*, light green). Neurons within the low-amplitude clusters exhibited strong responses to low-stimulation amplitudes (20–40% MT), but not higher stimulation amplitudes (60–80% MT). We quantified the proportion of neurons in each cluster across stimulation frequency (50- vs 90-Hz Aβ-ES; Fig. 8*C*,*D*), pain condition (none vs bicuculline), and neuron classification (pEX vs pIN; Fig. 6*B*).

While clustered responses to stimulation amplitude were heterogeneous across all conditions, several key differences in response clusters emerged between stimulation parameters and pain conditions. First, 50- and 90-Hz Aβ-ES had similar effects on both pEX and pIN neurons when we examined changes with two clusters (Fig. 8*C*). However, after application of bicuculline, 90 Hz was more likely than 50 Hz to inhibit pEX neurons and excite pIN neurons. This difference in inhibition of pEX neurons was mostly driven by a decrease (−28%) in the proportion of pEX neurons that experienced monotonic excitation with 90-Hz stimulation. The difference in inhibition of pIN neurons was driven by a decrease in monotonic inhibition with 90-Hz stimulation (−15%). When comparing postbicuculline recordings to control recordings, 50-Hz Aβ-ES inhibited a larger proportion of pIN neurons (19%) but almost no additional pEX neurons (3%, two clusters; Fig. 8*D*). On the other hand, 90 Hz inhibited a much larger proportion of pEX neurons (17%) than pIN neurons (9%) after bicuculline. The increase in inhibition of pEX neurons after bicuculline comprised increases to both monotonic inhibition (+13%) and nonmonotonic low amplitude inhibition (+4%) responses. Overall, these results demonstrate that while *in vivo* responses to stimulation were heterogeneous, there was an interaction between stimulation parameters (frequency and amplitude) and the net inhibition of DH neurons. Additionally, local application of bicuculline produced significant changes to the responses of both pEX and pIN neurons with both 50 and 90 Hz, further implicating a segmental GABAergic mechanism (Fig. 6). Nonetheless, 90-Hz Aβ-ES remained effective at inhibiting DH neurons suggesting mechanisms beyond segmental GABAergic inhibition.

## Discussion

We hypothesized that recently observed low-rate (<200 Hz) subperception SCS (Metzger et al., 2021) produced rapid-onset pain relief by sparse activation of DC axons, which engaged surround inhibitory mechanisms in the DH. DC axons exhibited irregular patterns of activity during low amplitude SCS both *in silico* and *in vivo*. When applied as inputs to a validated DH network model, DC axon activity from low amplitude SCS maximized inhibition of both model WDR and pEX neurons, corroborating the potential role of DC activity in mediating pain relief. Furthermore, the responses of both model neurons and *in vivo* DH neurons were strongly dependent on the spatial location of stimulation with respect to the “center” painful receptive field, supporting a mechanistic role for surround inhibition. The degree to which SCS inhibited the excitatory neurons was dependent on stimulation parameters, and some paradigms (e.g., low-amplitude, 90-Hz stimulation) produced consistent inhibition across pain states. Blocking local GABAergic inhibition with bicuculline affected neuronal responses, corroborating a role for a segmental GABAergic mechanism, and stimulation frequency had a strong effect on net inhibition, even during low amplitude stimulation. Collectively, these results implicate surround inhibition as a driver of the effects of rapid-onset, low-rate subperception SCS and provide insight into possible strategies for further optimizing SCS.

### Surround inhibition

The strong net inhibitory effect of low amplitude stimulation depended on the location where DH neurons were recorded. Tibial Aβ-ES that primarily activated the L4 and L5 nerve roots produced strong inhibition of neurons at L3 compared with mixed responses at L4 and excitation at L5 (Swett et al., 1991). These responses are consistent with previous recordings of DH neurons that demonstrated inhibition from electrical stimulation of a nerve from a different area of the receptive field (Hillman and Wall, 1969; Foreman et al., 1976). Additionally, Aβ-ES of nerve roots adjacent to C-fiber stimulation was much more likely to produce inhibition in both excitatory interneurons and Lamina I projection neurons than Aβ-ES at the same root as C-fiber stimulation (Luz et al., 2014; Fan and Sdrulla, 2020). The striking parallels between the effects of spatial targeting in the computational model (Fig. 5*C*) and the spatial distribution of responses of pEX neurons to Aβ-ES *in vivo* (Fig. 7*C*) demonstrates the importance of considering and exploiting the spatial organization of inputs to the DH (Hillman and Wall, 1969). Additionally, these spatial effects were specific to pEX neurons and pIN neurons did not have the same striking differences in responses based on their spatial distribution (Extended Data Fig. 7-1).

Our results suggest that activation of afferents from surround receptive field areas is important for achieving pain relief, while clinical reports indicate that a high degree of overlap between the pain area and paresthesia area is required for pain relief. However, these are not necessarily contradictory observations because of topographic differences between pain and paresthesia sensations. Crush stimulation preferentially activated pEX neurons in the rostral zones (Fig. 7*E*), consistent with previous descriptions of a rostral bias in the strength and location of Aδ and C-fiber inputs to Lamina I projection neurons (Pinto et al., 2010; Fernandes et al., 2016). Unlike crush stimulation, brushing the hindpaw facilitated pEX neuron activity regardless of recording position. The difference in spatial responses between Aβ-ES and crush suggest somewhat distinct somatotopic representations of pain and paresthesia sensations within the dorsal horn and may also be explained by the more extensive Aβ fiber collateralization versus Aδ and C fiber collateralization (Kuehn et al., 2019).

### Importance of stimulation parameters

In contrast to higher amplitude SCS, SCS applied just above activation threshold generated irregular patterns of DC axon activity (Fig. 4). These activity patterns reduced WDR firing rates in the DH network model more so than DC axon activity synchronized with the stimulation train, predicting that stimulation amplitude plays a role in the mechanisms of action SCS. SCS applied at amplitudes as low as 60% of the predicted sensory threshold (30% of predicted MT) activated model DC axons, and subsequently reduced model WDR firing rates across all tested frequencies. However, changes in WDR firing rate depended nonmonotonically on both stimulation amplitude and frequency. Maximum suppression occurred at 75–85% of sensory threshold (40% of predicted MT) in the computational model, and 50–90 Hz led to greater inhibition of WDR neurons over a broader range of amplitudes than other frequencies (Fig. 5). This matches with clinical observations of pain relief with subperception SCS applied at a frequency of 90 Hz and pulse width of 210 ± 50 μs (Metzger et al., 2021).

The lack of paresthesia evoked by low-amplitude, low-frequency SCS may be because of desynchronized activity, as only few axons faithfully follow stimulation. A previous study found that constant stimulation produced paresthesia that felt unnatural, but modulation of stimulation pulse width during stimulation produced changes in firing rate across the target population that did not evoke paresthesia (Tan et al., 2014). In our computational model, almost no axons fired at the stimulation frequency ≤110% of their AT, and this increased to only ∼50% at 120% of the AT (Fig. 4*A*). This desynchronized activity was consistent with our *in vivo* recordings of DC axons (Fig. 4*B*) and may underlie inhibition without paresthesia. Additionally, a human scale patient-specific model of SCS found remarkable similarity between absolute values of clinical sensory thresholds and model predicted thresholds using the assumption that the sensory threshold occurred when >10% of the model DC axons were activated (Lempka et al., 2020). Supporting DC activation during low-amplitude SCS, prior studies recorded evoked compound action potentials (ECAPs) from the DCs below 50% of MT (or 100% of PT) in a preclinical model (Yang et al., 2015), and clinical reports with closed-loop SCS showed that ECAPs occur below sensory threshold in some patients (Pilitsis et al., 2021). A study of continuous 50-Hz subperception SCS found decreased mechanical hypersensitivity and altered theta rhythms in awake freely moving rats (Koyama et al., 2018). Finally, electroencephalography (EEG) recordings during electrical stimulation below PT indicated that stimulation can have significant direct effects on somatosensory responses without evoking perception (Iliopoulos et al., 2020).

### DH neuron responses following disinhibition

Neuropathic pain leads to disinhibition in DH networks through a variety of mechanisms (Sandkuhler, 2009). Applying different modes of disinhibition to the network model increased baseline firing rates but did not change the overall trend of responses to SCS (Fig. 5). Responses to SCS were more variable across different neuropathic pain states, and higher stimulation amplitudes led to greater relative inhibition of WDR neurons than in the healthy model conditions. Additionally, optimal stimulation parameters depended strongly on stimulation conditions and showed greater heterogeneity compared with naive conditions (Fig. 5*H*), indicating that additional patient-specific mechanisms, or computational models personalized to individual patients, may be necessary to optimize stimulation (Lempka et al., 2020).

Following application of bicuculline *in vivo*, there was a significant increase in pEX neuron spontaneous activity and responses to brush (Fig. 8). Receptive fields of pEX neurons expand following disinhibition, thus unmasking additional excitation and reducing some of the effects of surround inhibition (Lee et al., 2019). Similarly, we observed a substantial shift of neurons to the low threshold excitatory cluster following bicuculline and decreased inhibitory effect from lower amplitudes of Aβ-ES on pEX neuron activity, suggesting a GABAergic contribution to the segmental effects of surround inhibition. Prior studies also demonstrated reduced inhibition of projection neurons from SCS following application of bicuculline (Duggan and Foong, 1985; Zhang et al., 2015), and chronic pain models demonstrated reduced GABAergic inhibition compared with glycinergic inhibition (Moore et al., 2002). The partial rescue of inhibitory effects by increasing stimulation amplitude supports recruitment of additional inhibitory mechanisms, e.g., glycinergic neurons (Braz et al., 2014), but inhibition remained impaired.

### Limitations

We used a previously published classification system to segregate neural types in our recordings (Lee et al., 2019), but classification based on waveform shape is a relatively new method and may miss some of the heterogeneity within DH populations. In our study, pIN neurons were generally suppressed by stimulation, which goes against what we would expect to find with gate-control SCS. However, pIN neurons exhibited a wide array of responses, and pIN neurons that were inhibited by stimulation may be a part of other neural microcircuits that are quite complex (Prescott and Ratte, 2012; Prescott, 2015). Second, to enable receptive field targeted stimulation, we delivered Aβ-ES to a peripheral nerve, and future experiments should explore effects of spatial targeting with DC stimulation. Third, the amplitudes for effective subperception SCS (70–100% PT) were higher than those reported in a clinical study of subperception low-rate SCS (20–70% PT; Metzger et al., 2021). However, this could be because of differences between rat and human anatomy, or because motor thresholds are an inexact method for determining stimulation amplitude (Koyama et al., 2018). Also, we correlated model WDR projection neuron activity with *in* vivo responses but did not test whether any neurons were projection neurons; however, we did identify neurons based on their responses to mechanical stimulation. Nonetheless, interneurons play vital roles in gating, integrating, and relaying sensory and nociceptive information through the dorsal horn (Prescott et al., 2014). Finally, our *in vivo* experiments were only performed on male rats, but previous studies have demonstrated conflicting effects of sex on responses to chronic pain (Coyle et al., 1995; Tall et al., 2001; LaCroix-Fralish et al., 2005), and other preclinical studies (Song et al., 2009; Barchini et al., 2012) and clinical studies (Kumar and Toth, 1998; Mekhail et al., 2022) have found comparable outcomes to SCS between males and females. Therefore, we did not include sex-based differences in our study design, but this may be addressed with future studies.

We made several important assumptions with the computational models. First, specific connections within the network model were based on descriptions of DH recordings and prior models, but specific synaptic conductances are difficult to match. Also, the model did not include the effects of supraspinal inputs, and although a role for supraspinal inputs for conventional SCS that produces paresthesia has been established (Saade et al., 2015; Linderoth and Foreman, 2017), the role of descending modulation during subperception SCS remains unclear. Prior studies of DH circuits indicate that there is significant variability within DH neural populations, but we only accounted for a single neuron for each type in the model. We also assumed that the DCs were the only elements activated by stimulation and ignored potential direct field effects on individual neurons, although previous computational modeling studies have found that the activation thresholds of DH neurons are always higher than DC axons (Rogers et al., 2022).

## Synthesis

Reviewing Editor: Niraj Desai, National Institute of Neurological Disorders and Stroke Decisions are customarily a result of the Reviewing Editor and the peer reviewers coming together and discussing their recommendations until a consensus is reached. When revisions are invited, a fact-based synthesis statement explaining their decision and outlining what is needed to prepare a revision will be listed below. The following reviewer(s) agreed to reveal their identity: Andrei Sdrulla.

Your manuscript has now been read carefully by two reviewers. Both were impressed by the work, as was I, but both also offered extensive comments (see below). One common sentiment was that the manuscript, especially given the mix of modeling and experiment, was sometimes difficult to follow. This doesn’t require extensive rewriting, but it might require some reorganization to make sure the reader follows.

REVIEW #1

This manuscript investigated the effects of peripheral and dorsal column stimulation on axonal activation and dorsal horn neuronal activity. The authors found that 90 Hz stimulation drove heterogeneous activation of DC axons when stimulating using amplitudes close to the activation threshold. Using modeling and in vivo recordings, they found that sub paresthesia threshold stimulation at 90 Hz significantly depressed neuronal activity when delivered to axons from the surround field. Other parameters were less effective at suppressing activity. Finally, the authors identified both GABAergic dependent and independent mechanisms for the observed effects.

This is an important study that will be of interest to the pain neuroscience and neuromodulation communities as it explores the biological mechanisms of spinal cord stimulation, a modality commonly used to treat refractory pain.

Enthusiasm is diminished by the mixed in vivo/modeling approaches, making the manuscript challenging to follow and sometimes confusing.

1. The key finding that DC and Abeta stimulation suppresses the activity of pIN neurons is extremely surprising as one would have expected differential effects, with suppression of pEX neurons and activation of pIN neurons. The observation that they are both suppressed is counter to gate control theory. If pIN are also inhibited, what is driving the surround inhibition?

2. Why was 90 Hz stimulation used? The authors mention a clinical study, but this is not a sufficient reason for choosing those parameters. Was 90 Hz/225 superior to the other combinations (Figure 5) in suppressing WDR activity?

3. What was the depth of recording of DH neurons? The methods should include that information as it is pertinent to the laminar location of the neurons. Given that brush activated pEX neurons, it is likely that the recordings were from deeper laminae.

4. Where some of the neurons recorded WDR? If so, did they fall in the excitatory neuron category? This would support the classification criteria used by the authors.

5. The results shown in Figure 3 are mentioned in the methods. It should be changed to be included in the Results section.

6. The first paragraph of the results section should be moved to the discussion.

7. Where is the stimulation artifact for DC axon recordings (Figure 3c)? How was the artifact removed for experiments such as those described in Figure 6-1?

8. There needs to be better clarification as to how long stimulation was delivered.

9. Why did fewer neurons respond to Abeta stimulation (Figure 3-1) after the application of bicuculline? Multiple prior studies have shown that disinhibition uncovers Abeta drive into excitatory circuits, whereas here, it depressed pEx activity.

10. Please include the data for pIN in Figure 7.

Minor editing:

Page 19, last line “responses. while"

Why are there boxes/lines around graphs in Figure 3-1, 8?

Page 24, last paragraph, second line “(Error!...”

REVIEW #2

The manuscript “Surround Inhibition Mediates Pain Relief by Low Amplitude Spinal Cord Stimulation: Modeling and Measurement” presents technically complex and multi facet study focused on mechanisms underlying subperception spinal cord stimulation (SCS) for treating chronic neuropathic pain. The authors explore hypothesis that surround inhibition provided by surround receptive fields support low frequency low amplitude SCS. The reviewer highly values the parallel nature of the study combining modeling and electrophysiological measurements. The manuscript is clearly written but appears to be succinct to the extent that it is hard to follow the evidence presented in the support of the authors’ hypothesis.

1. The model would be hardly possible to reproduce following the instructions of the manuscript since the details are distributed over more than three sources. I would suggest that authors provide at least properties of the neurons in the circuitry and properties of the synaptic connections. Could authors comment on resonance properties of these neurons?

2. Synchronization plays important roles in this study. What is/are the measure(s) of the synchronization used by authors? How are membrane and synaptic properties distributed over the network? Could authors comment on neuronal spiking properties, are they integrate-and-fire or resonate-and-fire? How many model networks were generated and investigated?

3. Model figures provide many interesting results but the results section describes them sparsely, rather providing conclusions than their description.

4. Recordings are made from male rats. Could authors discuss why results were not obtained for female rats as well?

Minor

The figure 1 describing the models of DC axons referred to [34] and the finite element method model of the rat spinal cord referred to [44]; and Figure 2 and Figure 5 cite [55], which could not be found in references since those are not numbered.

"Code required to reproduce the figures in this manuscript will be made available.” Where? What type of code is it?

## Author Response

Synthesis of Reviews:

Computational Neuroscience Model Code Accessibility Comments for Author (Required):

In their resubmission, the authors should describe the code more thoroughly (language? system requirements?). Even better is if they provided the code to the reviewers to examine.

Code was uploaded for access by the Reviewers. Code required to reproduce figures (MATLAB) will be available on request and code for model (NEURON) will be posted on ModelDB).

Significance Statement Comments for Author (Required): N/A

Comments on the Visual Abstract for Author (Required): N/A

Synthesis Statement for Author (Required): Your manuscript has now been read carefully by two reviewers. Both were impressed by the work, as was I, but both also offered extensive comments (see below). One common sentiment was that the manuscript, especially given the mix of modeling and experiment, was sometimes difficult to follow. This doesn’t require extensive rewriting, but it might require some reorganization to make sure the reader follows.

REVIEW #1

This manuscript investigated the effects of peripheral and dorsal column stimulation on axonal activation and dorsal horn neuronal activity. The authors found that 90 Hz stimulation drove heterogeneous activation of DC axons when stimulating using amplitudes close to the activation threshold. Using modeling and in vivo recordings, they found that sub paresthesia threshold stimulation at 90 Hz significantly depressed neuronal activity when delivered to axons from the surround field. Other parameters were less effective at suppressing activity. Finally, the authors identified both GABAergic dependent and independent mechanisms for the observed effects.

This is an important study that will be of interest to the pain neuroscience and neuromodulation communities as it explores the biological mechanisms of spinal cord stimulation, a modality commonly used to treat refractory pain.

Enthusiasm is diminished by the mixed in vivo/modeling approaches, making the manuscript challenging to follow and sometimes confusing.

1. The key finding that DC and Abeta stimulation suppresses the activity of pIN neurons is extremely surprising as one would have expected differential effects, with suppression of pEX neurons and activation of pIN neurons. The observation that they are both suppressed is counter to gate control theory. If pIN are also inhibited, what is driving the surround inhibition?

We agree with the Reviewer that the finding that pIN neurons are suppressed by stimulation is initially surprising and potentially at odds with the gate control theory. However, in our in vivo studies, we recorded from a wide range of mechanically responsive DH neurons, and there was substantial heterogeneity in the responses across the neuron classes. Therefore, pIN neurons that were excited by stimulation may be classic inhibitory gate-control neurons, while pIN neurons that did not respond to stimulation may be part of other neural circuits (e.g., feedback inhibition). Dorsal horn microcircuits are complex (Prescott & Ratte, 2012), and inhibitory neurons often target other inhibitory neurons (Prescott, 2015). Thus, the pIN neurons that were inhibited by stimulation may not be a part of the classic gate control microcircuits. As stated in the limitations of the revised manuscript, our classification system “is a relatively new method and may miss some of the heterogeneity within DH populations.”

We updated the Discussion to reflect the important point the Reviewer raised and included additional comments about the heterogeneous responses of pIN neurons (line 612), “In our study, pIN neurons were generally suppressed by stimulation, which is contrary to what one would expect from gate-control theory. However, pIN neurons exhibited a wide array of responses, and pIN neurons that were inhibited by stimulation may be a part of other microcircuits that are quite complex (Prescott, 2015; Prescott & Ratte, 2012).”

2. Why was 90 Hz stimulation used? The authors mention a clinical study, but this is not a sufficient reason for choosing those parameters. Was 90 Hz/225 superior to the other combinations (Figure 5) in suppressing WDR activity?

The Reviewer raises an important point about stimulation parameters. We did indeed find that 90 Hz/225 μs generated the most inhibition in 11 of the 25 random conditions we tested (Figure 5), i.e., this was the most effective combination. Together with the fact that this was the parameter combination used in the clinical study that inspired this work (Metzger et al., 2021), this was adequate justification for choosing this parameter combination.

We updated the Results (line 395) to include an additional sentence that these simulations provided additional justification for our in vivo tests, “That 90 Hz stimulation was optimal in the most model conditions and that 90 Hz stimulation was used during the clinical study of fast-acting subperception SCS justified the use of 90 Hz stimulation for our in vivo experiments (Metzger et al., 2021).”

3. What was the depth of recording of DH neurons? The methods should include that information as it is pertinent to the laminar location of the neurons. Given that brush activated pEX neurons, it is likely that the recordings were from deeper laminae.

We updated the Methods to clarify the range of recording depths (line 222) reads, “16-contact electrodes had a recording span of 375 μm (Neuronexus electrode A1x16-Poly2-5mm-50s-177-A16) and 32-contact electrodes had a recording span of 275 μm (Neuronexus electrode A1x32-Poly3-5mm-25s-177). Electrodes were inserted at a 30-45{degree sign} rostral-caudal and medial-lateral angle just medial to the dorsal root entry zone. Electrode depths spanned from approximately 50 μm to 800 μm with an average depth of 400 μm.”

4. Where some of the neurons recorded WDR? If so, did they fall in the excitatory neuron category? This would support the classification criteria used by the authors.

We agree with the Reviewer that mechanical classification of neurons is important as well. We included a statement in Methods indicating the proportion pIN and pEX neurons that were also classified as WDR neurons. Line 257 in Methods, “Among neurons classified as pEX that we identified as mechanically responsive, 12% were classified as high threshold (only responded to crush), 76% were wide-dynamic range neurons (increasing response rates with increasing stimulus strength), and 12% were low-threshold (only responded to brush and/or press). Among neurons classified as pIN that were mechanically responsive, 18% were high threshold, 55% were wide-dynamic range, and 27% were low-threshold.”

5. The results shown in Figure 3 are mentioned in the methods. It should be changed to be included in the Results section.

Figure 3 highlights the methods used for other figures in the Results. However, it only includes data from a single recording as an example for the rest of the figures in the Results. Therefore, we feel it is more appropriate to only include these results in the Methods. We updated the description to clarify that Figure 3F is only an example of generating PSTHs for later in the Results.

6. The first paragraph of the results section should be moved to the discussion.

The first paragraph of the Results provides a broad overview of the results section, and we feel this is particularly important given the breadth of the data, and this helps to address the Reviewers’ concern about the difficulty in following the combination of both modeling and experimental work.

7. Where is the stimulation artifact for DC axon recordings (Figure 3c)? How was the artifact removed for experiments such as those described in Figure 6-1?

The stimulation artifact in Figure 3c is approximately 0.5 ms wide and occurs approximately 1 ms before the start of each action potential. Therefore, it cannot be visually distinguished from the action potential in the longer time base view, and it was specifically left out of the zoomed window of the action potential waveform. The artifact was not removed during action potential detection; however, only units which could be readily distinguished from the artifact were included in the analysis. A sentence was added to the Methods to describe the artifact (line 202), “The stimulation artifact was approximately 0.5 ms wide and occurred approximately 1 ms before the start of each action potential; the artifact cannot be distinguished from individual action potentials in Figure 3c and was removed from the zoomed in window.”

8. There needs to be better clarification as to how long stimulation was delivered.

We revised the Methods to include a statement about the duration of stimulation for both DC axon and DH neuron recordings. For DC axon recordings (line 199), “Each stimulation block for DC axon recordings lasted approximately 4 minutes, and units were reconfirmed between stimulation blocks.” For DH neuron recordings (line 243), “Each stimulation block lasted 11 minutes with ∼20-30 minutes before each block to record spontaneous activity and responses to mechanical stimulation. Units were confirmed through online sorting prior to each stimulation block.”

9. Why did fewer neurons respond to Abeta stimulation (Figure 3-1) after the application of bicuculline? Multiple prior studies have shown that disinhibition uncovers Abeta drive into excitatory circuits, whereas here, it depressed pEx activity.

The Reviewer raises an important point for clarification. Extended Figure 3-1 indicates that fewer neurons responded to stimulation following application of bicuculline. The overall firing rates of pEX neurons increased after application of bicuculline, but fewer were classified as responders to stimulation after bicuculline than in the control condition. We revised the Results and figure caption to reflect this clarification. In results (line 432), “The proportion of pEX but not pIN neurons responding to Aβ-ES (i.e. exhibiting a significant change in activity during stimulation compared to pre-stimulation) was substantially reduced by application of bicuculline compared to control (non-bicuculline) recordings, supporting a segmentally mediated but complex GABAergic mechanism as a driver for low-amplitude Aβ-ES mediated inhibition (pEX: 53% responders to stimulation in control recordings versus 24% in bicuculline recordings, pIN: 49% control vs. 52% bicuculline, Extended Figure 3-1).”

For figure caption, “Responders indicate neurons that exhibited a significant change in activity quantified by their PSTH for during stimulation compared to pre-stimulation.”

10. Please include the data for pIN in Figure 7.

The data for pIN neurons are included in Figure 6b1 and b2. However, pIN neurons did not exhibit significant spatial differences so we did not include them in Figure 7.

We revised the manuscript to include a statement that pIN neuron responses were not significantly different across recording positions and included pIN neurons in extended figure 7-1.

In Results (line 456), “However, responses for pIN neurons were not dependent on position (ANOVA, p = 0.15, Extended Figure 7-1).”

In Discussion (line 540), “Additionally, these spatial effects were specific to pEX neurons and pIN neurons did not have the same striking differences in responses based on their spatial distribution (Extended Figure 7-1).”

Extended Figure 7-1 Caption, “Extended Figure 7-1: DH responses to Aβ-ES for pIN neurons do not depend on receptive field targeting. A) Individual DH neuron responses were sorted into three groups based on the location where they were recorded. C) False color maps of changes in pIN neuron activity vs. baseline activity split by recording position. D) Same as C, but raw firing rate changes vs. baseline. In C and D, data are from pIN neurons classified as responders by recording location, and the gray dotted lines between 40% an 60% MT represent estimated PT.”

Minor editing: Page 19, last line “responses. while" corrected

Why are there boxes/lines around graphs in Figure 3-1, 8?

The boxes around the graphs in Extended Figure 3-1 were apparently a file formatting issue and were corrected.

The box in Figure 8 shows that the clusters in boxes 2 and 4 are the optimal distribution for clustering the responses based on quantitative metrics, and the caption in Figure 8 was updated to clarify these boxes, “Quantitative analysis identified either two (Davies & Bouldin, 1979) or four (Rousseeuw, 1987) as the optimal number of clusters, and boxes were placed around these plots.”

Page 24, last paragraph, second line “(Error!...” corrected

REVIEW #2

The manuscript “Surround Inhibition Mediates Pain Relief by Low Amplitude Spinal Cord Stimulation: Modeling and Measurement” presents technically complex and multi facet study focused on mechanisms underlying subperception spinal cord stimulation (SCS) for treating chronic neuropathic pain. The authors explore hypothesis that surround inhibition provided by surround receptive fields support low frequency low amplitude SCS. The reviewer highly values the parallel nature of the study combining modeling and electrophysiological measurements. The manuscript is clearly written but appears to be succinct to the extent that it is hard to follow the evidence presented in the support of the authors’ hypothesis.

1. The model would be hardly possible to reproduce following the instructions of the manuscript since the details are distributed over more than three sources. I would suggest that authors provide at least properties of the neurons in the circuitry and properties of the synaptic connections. Could authors comment on resonance properties of these neurons?

We agree with the Reviewer that there are several different sources and models that contributed to this work. We will make available all code required to reproduce the figures in one location so that each component can be reproduced. We will also upload the code for access by the Reviewers and post the model code on NEURON ModelDB.

We did not quantify whether the neurons in our model have a specific frequency preference (i.e., resonance).

2. Synchronization plays important roles in this study. What is/are the measure(s) of the synchronization used by authors? How are membrane and synaptic properties distributed over the network? Could authors comment on neuronal spiking properties, are they integrate-and-fire or resonate-and-fire? How many model networks were generated and investigated?

We agree with the Reviewer that synchronization plays an important role in the study. Synchronization of DC axon spiking is directly addressed in Figure 4 which includes rasters of spiking responses (Fig 4a,b), summary metrics of the proportions of axons responding at particular frequencies (Fig 4c), and a summary of the proportion of axons that are synchronized with the stimulation pulses (Fig 4d) for both model and experiment. Synchronization plays a more indirect role in responses of model dorsal horn neurons, and we focused on rate based coding metrics because these are more commonly used for comparisons between stimulation parameters.

The model neurons are biophysically realistic representations with a complement of non-linear ionic conductances that were described extensively in prior work. We added a section in the Methods to provide additional information about the biophysical properties of the neurons in question (line 100), “The model had three types of neurons, an inhibitory (IN) interneuron, an excitatory (EX) interneuron, and a wide-dynamic range (WDR) projection neuron, and each model neuron contains four compartments: a dendrite, soma, axon hillock, and axon. We modeled ionic currents in each type of model neuron using Hodgkin-Huxley-like membrane models replicated from the prior model (Zhang et al., 2014).”

3. Model figures provide many interesting results but the results section describes them sparsely, rather providing conclusions than their description.

We agree with the Reviewer that some figures would benefit from additional descriptions in the text. We added additional text to the Results for figures 5, 6, and 8 to provide descriptions of the results. Added text is italicized.

For figure 5 (line 367), “In general, model WDR neurons were suppressed by stimulation relative to baseline activity, but model WDR neuron responses were dependent on the receptive field origin of activated DC axons (ANOVA, p<0.05, post-hoc Tukey’s test, p<0.05), i.e., the proportion of activated DC axons that arose from the surround receptive field area versus the center receptive field area.”

Line 385, “Increasing the frequency of SCS targeted to surround receptive fields increased the maximum reduction in WDR firing rate but decreased the range of amplitudes that produced a reduction in WDR firing rate. For example, the range of stimulation amplitudes that reduced the firing rate of WDR neurons by at least 50% was 35%-60% of MT for 50 Hz/300 μs stimulation, but only 35%-40% for 200 Hz/200 μs stimulation. Higher stimulation frequencies also resulted in excitation of WDR neurons at higher amplitudes of SCS (Figure 5f).”

Line 412, “We plotted the changes in activity of all neurons during stimulation and detected no differences in the distribution of inhibitory vs. excitatory neuron responses to Aβ-ES between the pEX and pIN neuron classes (Figure 6a-b, ANOVA, p = 0.9).”

Line 477, “Fuzzy c-means clustering of the principal components of each response identified heterogeneous response types across the population of recorded neurons. Responses were optimally divided into two clusters (Davies-Bouldin metric (Davies & Bouldin, 1979)) or four clusters (silhouette metric (Rousseeuw, 1987), boxes, Figure 8a), and each line represents the average response of each neuron within that cluster for different stimulation amplitudes (Figure 8a). Two clusters divided into “excited” (purple) or “inhibited” (green) responses. Neurons within the “excited” cluster increased net excitation in response to stronger stimulation amplitudes, and “inhibited” responses showed the inverse. Four clusters divided into “monotonic excited” (light purple), “low-amplitude excited” (dark purple), “low-amplitude inhibited” (dark green), and “monotonic inhibited” responses (light green, Figure 8a). Neurons within the low-amplitude clusters exhibited strong responses to low stimulation amplitudes (20-40% MT), but not stronger stimulation amplitudes (60-80% MT).

4. Recordings are made from male rats. Could authors discuss why results were not obtained for female rats as well?

The present study investigated the mechanisms of action of spinal cord stimulation to treat chronic pain. Several studies found conflicting effects of sex on responses to chronic pain. Two prior studies found that female rats developed greater hypersensitivity than male rats following nerve injury (Coyle et al., 1995; LaCroix-Fralish et al., 2005), but another study indicated that hypersensitivity was greater in male rats (Tall et al., 2001). These conflicting results make it difficult to separate the analgesic effects of SCS from changes across sex. Furthermore, several prior studies indicated that sex likely does not affect behavioral outcomes of SCS. First, analgesic changes in paw withdrawal thresholds from SCS were comparable in studies using only female versus only male rats ((Barchini et al., 2012) versus (Song et al., 2009)). Second, several clinical studies that evaluated the impact of sex on responses to SCS found that there were no sex-based differences in the analgesic effect of SCS (Kumar & Toth, 1998; Mekhail et al., 2021; Taylor et al., 2005). Therefore, we chose not to address potential sex-based differences in our study design.

We addressed this point with text added to the Discussion (line 627), “Finally, our in vivo experiments were only conducted on male rats, but previous studies demonstrated conflicting effects of sex on responses to chronic pain (Coyle et al., 1995; LaCroix-Fralish et al., 2005; Tall et al., 2001), and preclinical (Barchini et al., 2012; Song et al., 2009) and clinical studies (Kumar & Toth, 1998; Mekhail et al., 2021) found comparable outcomes to SCS between males and females. Therefore, we did not include sex-based differences in our study design.”

Minor

The figure 1 describing the models of DC axons referred to [34] and the finite element method model of the rat spinal cord referred to [44]; and Figure 2 and Figure 5 cite [55], which could not be found in references since those are not numbered.

These were corrected.

"Code required to reproduce the figures in this manuscript will be made available.” Where? What type of code is it?

Code will be provided to the reviewers and available on request. The code is a mix of Neuron and MATLAB code. Neuron for the actual modeling work and MATLAB for the analysis of experimental data.

References

Barchini, J., Tchachaghian, S., Shamaa, F., Jabbur, S. J., Meyerson, B. A., Song, Z., Linderoth, B., & Saade, N. E. (2012). Spinal segmental and supraspinal mechanisms underlying the pain-relieving effects of spinal cord stimulation: An experimental study in a rat model of neuropathy. Neuroscience, 215, 196-208.

Coyle, D. E., Sehlhorst, C. S., & Mascari, C. (1995). Female rats are more susceptible to the development of neuropathic pain using the partial sciatic nerve ligation (psnl) model. Neuroscience letters, 186(2), 135-138.

Davies, D. L., & Bouldin, D. W. (1979). A cluster separation measure. IEEE Transactions on Pattern Analysis and Machine Intelligence, PAMI-1(2), 224-227.

Kumar, K., & Toth, C. (1998). The role of spinal cord stimulation in the treatment of chronic pain postlaminectomy. Current Review of Pain, 2(2), 85-92.

LaCroix-Fralish, M. L., Rutkowski, M. D., Weinstein, J. N., Mogil, J. S., & DeLeo, J. A. (2005). The magnitude of mechanical allodynia in a rodent model of lumbar radiculopathy is dependent on strain and sex. Spine, 30(16).

Mekhail, N., Costandi, S., Saweris, Y., Armanyous, S., & Chauhan, G. (2021). Impact of biological sex on the outcomes of spinal cord stimulation in patients with chronic pain. Pain Practice, n/a(n/a).

Metzger, C. S., Hammond, M. B., Paz-Solis, J. F., Newton, W. J., Thomson, S. J., Pei, Y., Jain, R., Moffitt, M., L., A., & Doan, Q. (2021). A novel fast-acting sub-perception spinal cord stimulation therapy enables rapid onset of analgesia in patients with chronic pain. Expert Review of Medical Devices, 18(3), 299-306.

Prescott, S. A. (2015). Chapter twelve - synaptic inhibition and disinhibition in the spinal dorsal horn. In T. J. Price & G. Dussor (Eds.), Progress in molecular biology and translational science (Vol. 131, pp. 359-383): Academic Press.

Prescott, S. A., & Ratte, S. (2012). Pain processing by spinal microcircuits: Afferent combinatorics. Curr Opin Neurobiol, 22(4), 631-639.

Rousseeuw, R. J. (1987). Silhouettes: A graphical aid to the interpretation and validation of cluster analysis. Journal of computational and applied mathematics, 20, 53-65.

Song, Z., Ultenius, C., Meyerson, B. A., & Linderoth, B. (2009). Pain relief by spinal cord stimulation involves serotonergic mechanisms: An experimental study in a rat model of mononeuropathy. Pain, 147(1).

Tall, J. M., Stuesse, S. L., Cruce, W. L. R., & Crisp, T. (2001). Gender and the behavioral manifestations of neuropathic pain. Pharmacology Biochemistry and Behavior, 68(1), 99-104.

Taylor, R. S., Van Buyten, J.-P., & Buchser, E. (2005). Spinal cord stimulation for chronic back and leg pain and failed back surgery syndrome: A systematic review and analysis of prognostic factors. Spine, 30(1).

Zhang, T. C., Janik, J. J., & Grill, W. M. (2014). Modeling effects of spinal cord stimulation on wide-dynamic range dorsal horn neurons: Influence of stimulation frequency and gabaergic inhibition. J Neurophysiol, 112(3), 552-567.
