# Supplementary figures and images for: Surround Inhibition Mediates Pain Relief by Low Amplitude Spinal Cord Stimulation: Modeling and Measurement
Source: eNeuro. 2022 Oct 4;9(5):ENEURO.0058-22.2022. doi: 10.1523/ENEURO.0058-22.2022 (PMC9536854; doi:10.1523/ENEURO.0058-22.2022)

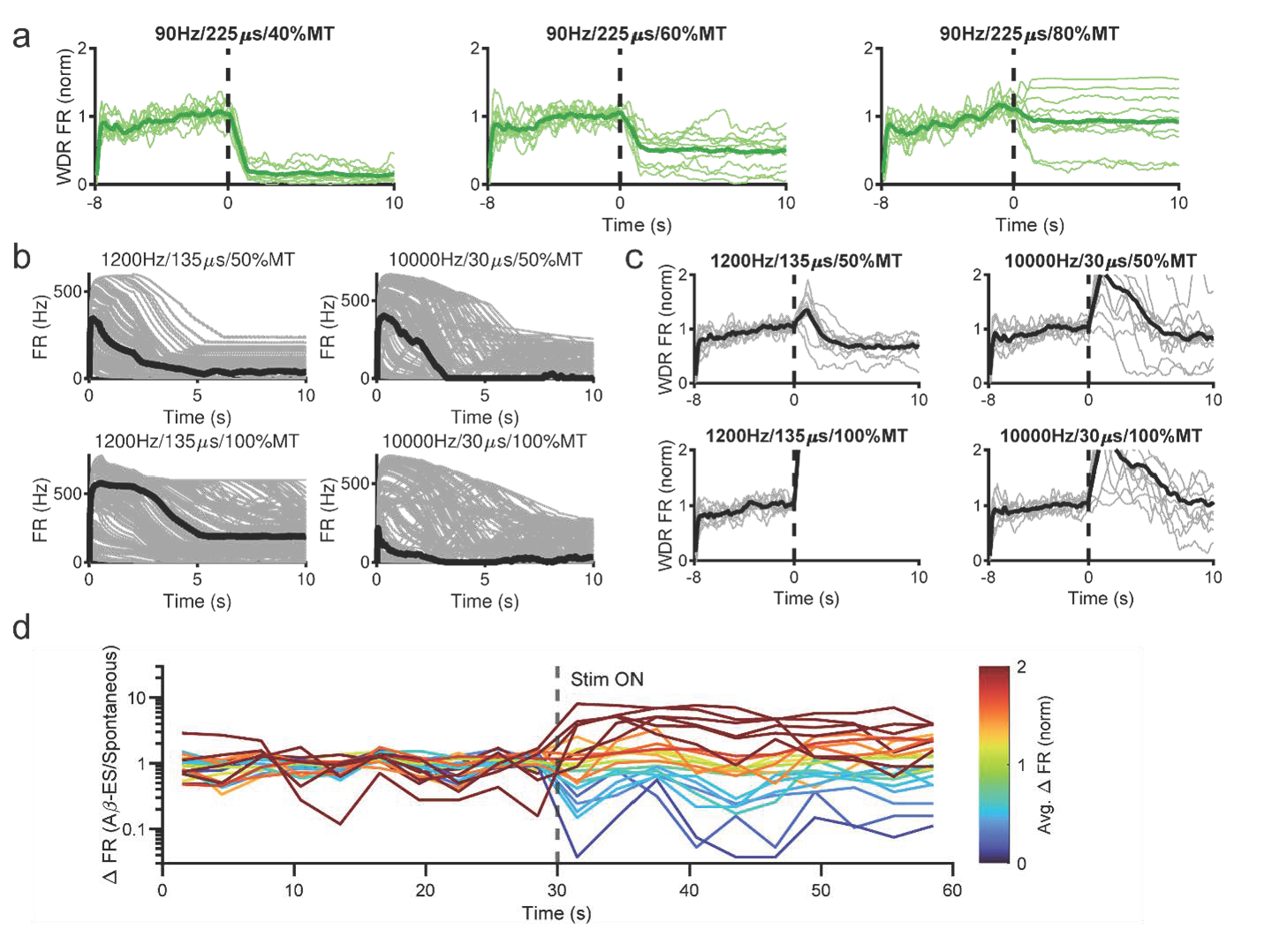

Supplement: Extended Data Figure 2-1 — Low-rate but not kilohertz frequency stimulation produces rapid-onset inhibition of DH neurons. A, Filtered firing rate of model WDR neurons. Each light line represents an individual response with a different map of randomly selected dorsal column axons. Dark lines represent median response. B, DC axon responses to kilohertz frequency stimulation. Light lines represent individual axon responses and dark line represents the median response. MT was estimated as 100 μA for 1200-Hz/135-μs stimulation and 300-μA/30-μs stimulation based on in vivo measurements in rats. C, Filtered firing rate of model WDR neuron responses to kHz frequency stimulation. D, Timescale of example trial from in vivo recordings of spontaneous activity (0–30 s) followed by Aβ-ES (30–60 s). Each line represents the filtered firing rate of an individual neuron. Colors represent the mean normalized change in firing rate across the 30-s window. Download Figure 2-1, TIF file. [file enu-eN-NWR-0058-22-s01.tif]

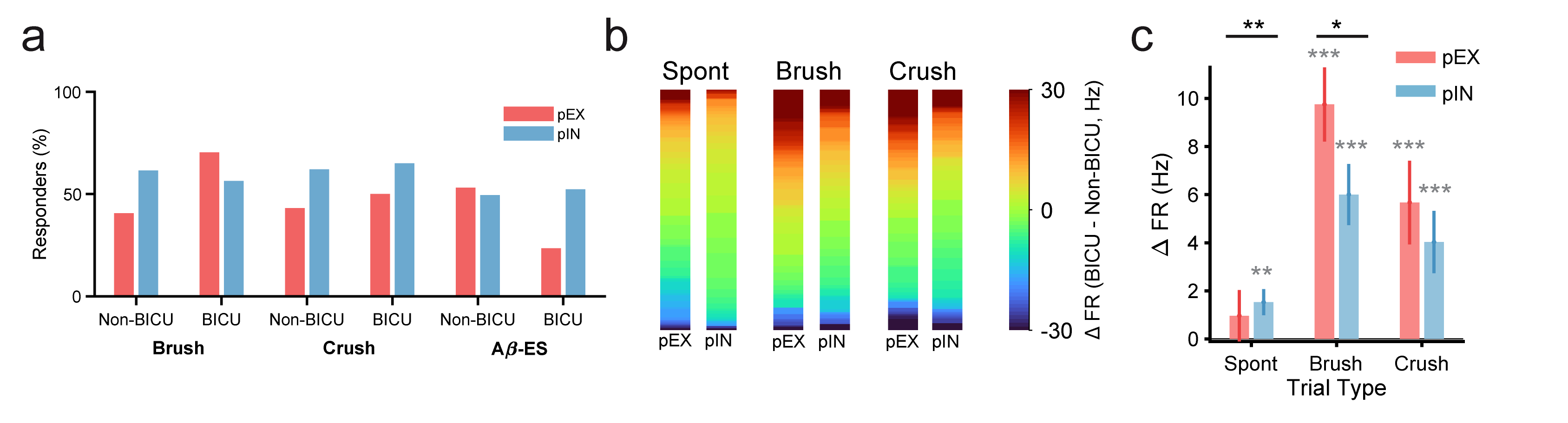

Supplement: Extended Data Figure 3-1 — Differential responses of DH neurons to mechanical stimulation before and after application of bicuculline support separate classification of pEX and pIN neurons. A, Percent of neurons that were responders to different stimulation modalities in recordings without bicuculline (non-BICU) and following application of bicuculline (BICU). Responders indicate neurons that exhibited a significant change in activity quantified by their PSTH for poststimulation compared to prestimulation. Neurons are split up by pEX or pIN waveform shape. B, Change in firing rate for spontaneous activity, brush responses, and crush responses following application of bicuculline. Colors represent the change from the non-BICU response to the BICU response. C, Mean change in firing rates across all neurons. Error bars are SE. Grey asterisks represent a significant change in responses (t test, *p < 0.05, **p < 0.01, ***p < 0.001). Black asterisks represent significant differences between pEX and pIN neurons (kstest2, *p < 0.05, **p < 0.01). Download Figure 3-1, TIF file. [file enu-eN-NWR-0058-22-s02.tif]

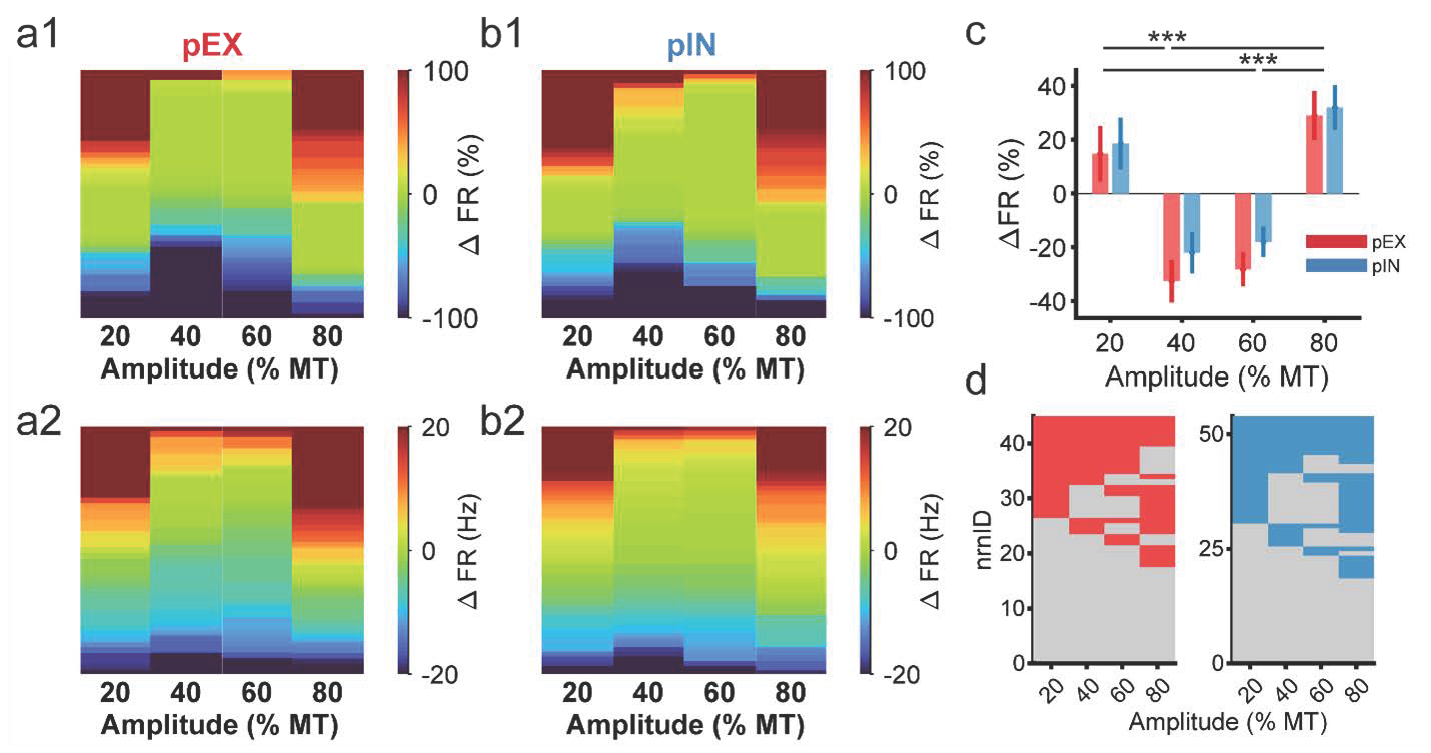

Supplement: Extended Data Figure 6-1 — Recorded neural responses to low-amplitude Aβ-ES at 90-Hz and 225-μs combined sciatic nerve stimulation at amplitude to activate C-fibers. A, Responses of pEX neurons in animals without bicuculline showing changes in firing rate normalized to the peak change in firing rate (A1) and raw changes in firing rate (A2). B, Same as A, but for pIN neurons. C, Mean normalized changes in pEX and pIN neuron responses to stimulation at different amplitudes. D, pEX (left) and pIN (right) neurons counted as responders for each stimulation amplitude. Colored boxes indicate neurons that are responders to stimulation. Error bars represent SE. Asterisks indicate significant difference between stimulation amplitudes (rmANOVA, p < 0.05, post hoc Tukey’s test, *p < 0.05, **p < 0.01, ***p < 0.001). Download Figure 6-1, TIF file. [file enu-eN-NWR-0058-22-s03.tif]

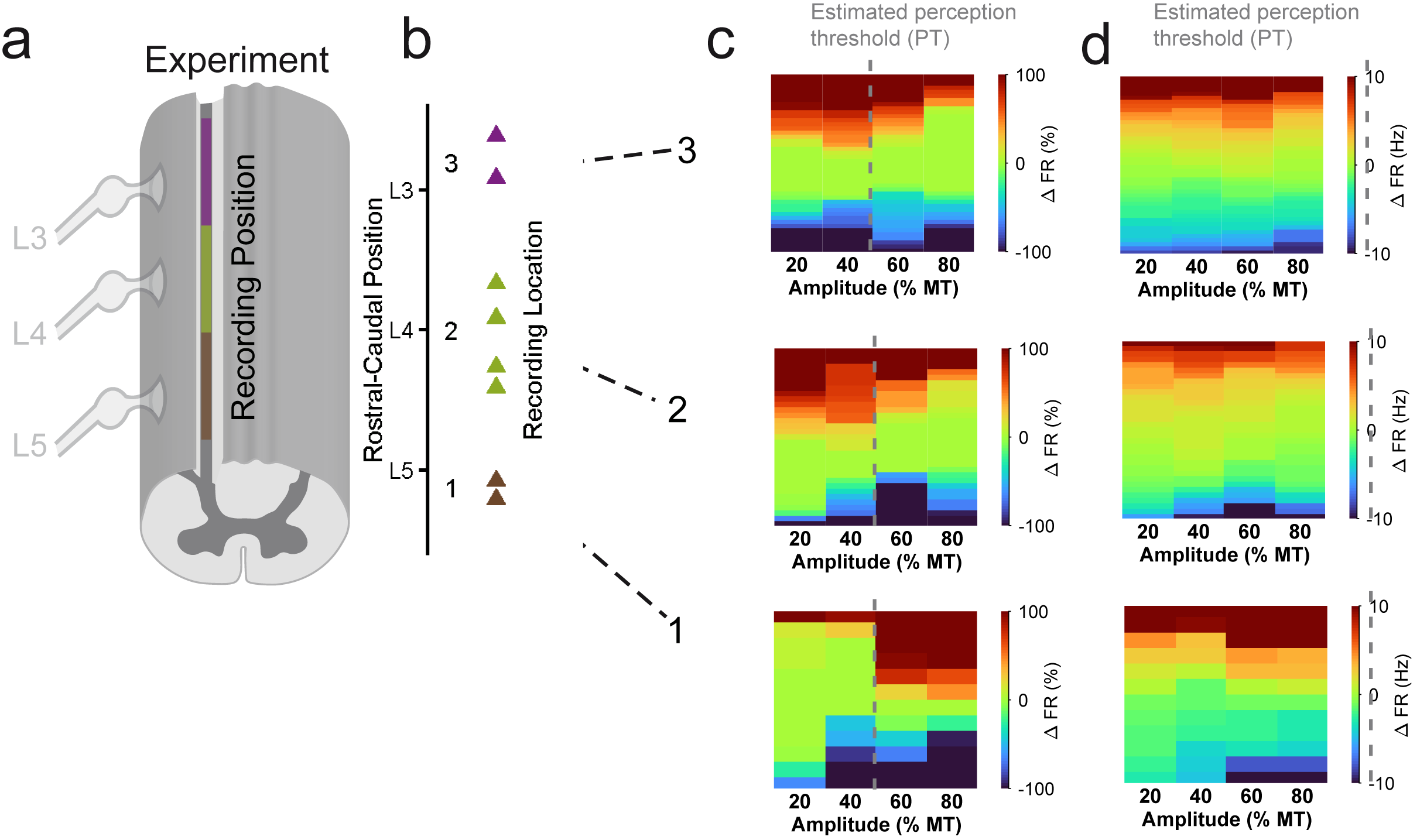

Supplement: Extended Data Figure 7-1 — DH responses to Aβ-ES for pIN neurons do not depend on receptive field targeting. A) Individual DH neuron responses were sorted into three groups based on the location where they were recorded. B) Normalized changes in pIN neuron activity divided by recording position. C) False color maps of changes in pIN neuron activity vs. baseline activity split by recording position. D) Same as C, but raw firing rate changes vs. baseline. In C and D, data are from pIN neurons classified as responders by recording location, and the gray dotted lines between 40% an 60% MT represents estimated PT. Download Figure 7-1, TIF file. [file enu-eN-NWR-0058-22-s04.tif]
